# Investigating the conformational landscape of human histidine-rich glycoprotein using amide HDX-MS

**DOI:** 10.1042/BCJ20253366

**Published:** 2025-11-17

**Authors:** Stephen J. Hierons, Boyang Lin, Remi Fritzen, Claudia A. Blindauer, Ramzi A. Ajjan, Glenn R. Masson, Alan J. Stewart

**Affiliations:** 1 School of Medicine, University of St Andrews, St Andrews, U.K.; 2 Department of Chemistry, University of Warwick, Coventry, U.K.; 3 Leeds Institute of Cardiovascular and Metabolic Medicine, University of Leeds, Leeds, U.K.; 4 Division of Cancer Research, School of Medicine, University of Dundee, Ninewells Hospital, Dundee, U.K.

**Keywords:** coagulation and fibrinolysis, conformational landscape, dynamics, haemostasis, histidine-rich glycoprotein, hydrogen-deuterium exchange mass spectrometry, intrinsic disorder, structural flexibility

## Abstract

Histidine-rich glycoprotein (HRG) is a multidomain plasma protein involved in immune modulation, angiogenesis, coagulation and fibrinolysis. Despite its broad biological relevance, structural investigations into HRG have yielded only limited information, with no experimentally resolved three-dimensional structures of the intact protein to date. In this study, we integrate hydrogen-deuterium exchange mass spectrometry (HDX-MS) with predictive insights from AlphaFold to map the conformational landscape of HRG in solution under near-native conditions. The N1/N2 domains displayed low solvent exchange overall. However, specific regions of high-solvent exchange were also apparent, providing evidence for more dynamic stretches of secondary structure and the presence of flexible loops within these regions. Our findings also reveal extensive solvent accessibility and rapid exchange kinetics within the histidine-rich region, proline-rich regions and large segments of the C-terminal domain, strongly indicating intrinsic disorder across these domains. These findings support a model in which structural flexibility underlies HRG’s capacity to engage with a wide range of molecular partners. This integrative approach offers new insight into the conformational architecture of HRG and lays the groundwork for uncovering the molecular mechanisms governing its biological activity.

## Introduction

Histidine-rich glycoprotein (HRG) is a soluble plasma protein that performs diverse functions and possesses unusual structural properties. HRG belongs to the type-III subgroup of the cystatin superfamily of cysteine protease inhibitors, the other members being fetuin-A/alpha-2-HS-glycoprotein, fetuin-B and kininogen [1]. The HRG domain structure consists of two N-terminal cystatin-like regions (N1 and N2), a central histidine-rich region (HRR), which is flanked by two proline-rich regions (PRRs), followed by a C-terminal domain (CTD) [1,2]. Of note, the HRR contains a highly conserved tandem repeat of the pentapeptide consensus sequence Gly-His-His-Pro-His [2,3]. HRG has 16 cysteine residues forming eight disulphide bonds, including three interdomain disulphides, with one of these linking the N1 and C-terminal domains, the second connecting the N2 domain to the HRR-PRR2 linker, and the third linking the HRR to the CTD [1,4]. Notably, human HRG is heavily glycosylated, with four N-linked glycosylation sites at Asn63, Asn125, Asn202 and Asn344. Additionally, O-linked glycans have been identified at Thr273 and Thr274 [5]. HRG has a broad interactome and has been shown to bind numerous targets including, but not limited to, fibrinogen, plasminogen, thrombospondin, IgG, complement factors, Fcγ receptors and glycosaminoglycans, as well as divalent metal ions [6–8]. Given its diversity of binding partners, HRG is implicated in several physiological processes, including antiangiogenic activity, immune complex clearance, pathogen clearance, coagulation and fibrinolysis [6–8].

There is growing interest in the role of HRG in the regulation of the coagulation and fibrinolysis pathways and how dysregulation of HRG-mediated haemostatic/fibrinolytic events may contribute to thrombotic disease [9,10]. HRG interacts with numerous haemostatic and fibrinolytic components including heparin/heparan sulphate, fibrinogen and plasminogen [6,7]. For instance, HRG binds activated factor 12 (FXIIa), which prevents its interaction with FXI and thus suppresses the intrinsic pathway of coagulation [11]. The complexation of HRG with heparin has been shown to limit heparin-mediated stabilisation of the antithrombin III-thrombin complex, which may represent an important mechanism for the pro-thrombotic properties of HRG [12,13]. HRG also binds fibrinogen and can be incorporated into fibrin clots, potentially generating thinner, more densely packed fibres, leading to restricted fibrinolytic capacity [14,15]. The interaction of HRG with plasminogen (and its subsequent effect on the fibrinolytic pathway) is of clinical interest, not least because activation of the fibrinolytic pathway is a key treatment modality for acute ischaemic stroke [16]. HRG has been shown to bind plasminogen with micromolar affinity and may be a potential plasma carrier of plasminogen *in vivo* [17]. When studied in solution, HRG inhibits fibrinolysis by sequestering plasminogen from its activating partner tissue plasminogen activator (tPA) [17,18]. However, upon HRR-mediated chelation of Zn^2+^, HRG localises to the cell surface where it acts as a receptor for plasminogen, resulting in enhanced plasminogen activation, which is predicted to enhance fibrinolysis [19,20]. HRG is proteolytically cleaved by plasmin at four putative cleavage sites at Lys275, Lys291, Arg339 and Arg439 [4,6,21]. Plasmin-cleaved HRG has been shown to have binding properties that are distinct from the intact protein, such as reduced ability to bind cell surface heparan sulphate, but an enhanced ability to bind necrotic cells and plasminogen [21]. Plasmin cleavage in combination with reduction, which is known to occur *in vivo* [4,6], yields a PRR/HRR peptide with anti-angiogenic properties [4,6,21]. Overall, all available evidence points towards a highly complex protein with vast functional capabilities, although the exact biological functions and mechanistic pathways remain incompletely understood.

Structural studies are essential to understand the mechanisms of protein function, but structural information for HRG is limited. Kassaar *et al*. have shown, using rabbit HRG, that N2 domains have a cystatin-like structure, composed of a 5-stranded antiparallel β-sheet, twisted around a five-turn α-helix [6]. *In silico* modelling suggests that HRG exhibits a large degree of conformational flexibility, with both the HRR and PRR regions predicted to be disordered (Supplementary Figure S1) [22–28]. Here, disorder is defined as a region which lacks compact secondary structure, with such regions being highly flexible and thus able to sample a wide range of conformational states. The presence of such regions within HRG likely contributes to its well-documented interaction promiscuity. Overall, a greater understanding of the HRG conformational landscape is needed to provide a structural framework for interpreting HRG’s diverse biological roles, including its involvement in coagulation and fibrinolysis. Given the likely inherent flexibility of HRG, a thorough structural investigation of this protein ultimately requires techniques that appropriately consider in-solution dynamics.

The aim of this current work is to further gain insights into the structure and dynamics of human HRG protein in solution using hydrogen/deuterium exchange mass spectrometry (HDX-MS). In HDX-MS, the rate of amide solvent exchange is measured via the incorporation of deuterium into the peptide backbone, with the rate of solvent exchange providing insight into protein structural dynamics [29]. Importantly, regions of the protein that are more flexible or exposed to the surrounding solvent tend to undergo deuterium exchange more rapidly, whereas areas that are buried or structurally constrained exhibit slower exchange [29]. HDX-MS provides peptide-level resolution and can therefore identify short stretches of both ordered and highly flexible structure within the intact protein. The ability to identify specific regions of high flexibility is particularly relevant to HRG, as such regions likely underpin its diverse interaction capabilities and are unlikely to be identified using other biophysical techniques. Overall, this approach offers a uniquely valuable, physiologically relevant view of the HRG structure’s topology.

## Results

### Secondary structure analysis of HRG using circular dichroism (CD)

**Figure 1:**
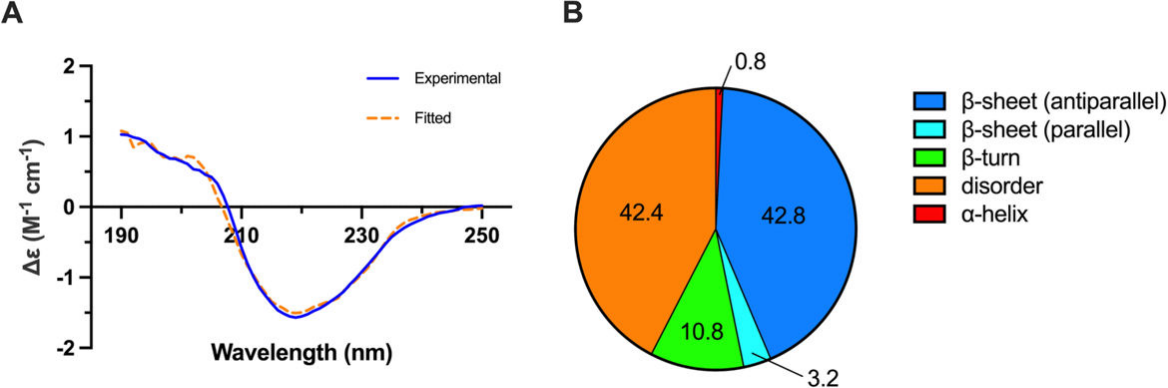
CD analysis of HRG. (A) Far UV CD spectrum of HRG. Far UV measurements used 0.2 mg/ml of HRG. HRG was prepared in 50 mM Tris, 140 mM NaCl buffer, pH 7.4. (B) Secondary structure content of HRG, as derived using the BeStSel algorithm [30].

To investigate its biophysical and structural properties, human HRG was isolated from human blood plasma using nickel-nitriloacetic acid (NTA) affinity chromatography as previously described [12]. Using this material, the secondary structure of plasma-derived human HRG was investigated by CD spectroscopy. Note, far ultraviolet (UV) CD data was collected between 180–250 nm; however, spectra acquired below 190 nm were deemed unreliable due to high signal-to-noise and were therefore omitted from analysis. The far UV CD spectrum of HRG indicated a significant degree of both β-sheet structure and disorder (Figure 1A). Deconvolution analysis using the BeStSel algorithm predicted that the protein consisted of 42.8% antiparallel β-sheet, 42.4% disorder, 10.8% β-turn, 3.2% parallel β-sheet and 0.8% α-helix (Figure 1B).

### Overcoming glycosylation challenges in liquid chromatography-tandem mass spectrometry (LC-MS/MS) analysis

**Figure 2:**
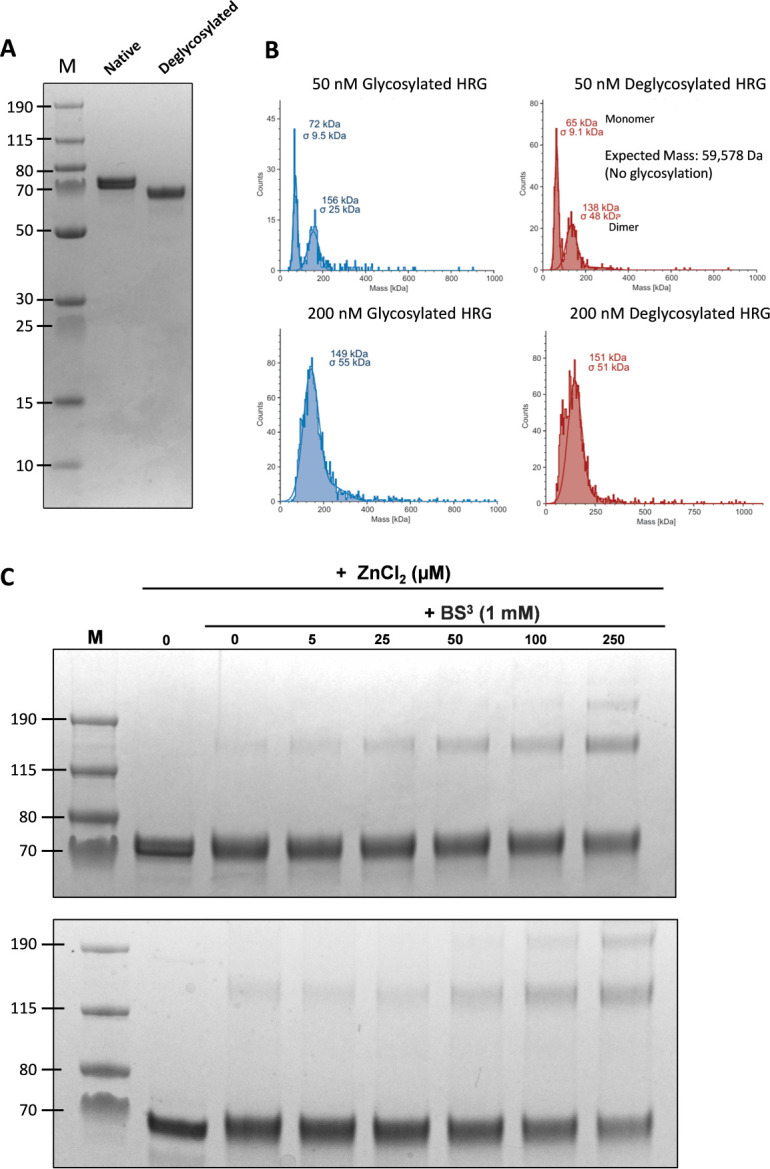
Deglycosylation of HRG and assessment of oligomeric properties. **(A**) SDS-PAGE gel of purified HRG before and after deglycosylation. HRG was deglycosylated under native conditions using the NEB protein deglycosylation mix II, which removes both N-glycans and some O-glycans. HRG was deglycosylated for 2 h at 37°C. (**B**) Solution-phase mass determination of native (blue) and deglycosylated (red) HRG samples using mass photometry. HRG samples were prepared at either 50 nM (top panels) or 200 nM (bottom panels) in 50 mM tris pH 7.4, 140 mM NaCl, 2 mM EDTA. (**C**) Effect of Zn^2+^ on HRG oligomerisation as assessed by chemical cross-linking using BS^3^. Here, native (upper panel) and deglycosylated (lower panel) HRG at 5 µM was incubated with 0, 5, 25, 50, 100 or 250 µM ZnCl_2_. BS^3^ was added to a final concentration of 1 mM. Reactions proceeded for 1 h at room temperature and were quenched via addition of 1 M Tris-HCl. Oligomers were identified by Coomassie-stained SDS-PAGE under reducing and denaturing conditions.

Heavily glycosylated proteins present several challenges to the HDX-MS workflow. In particular, the complexity and heterogeneity of the glycan structures significantly hinder the identification of glycopeptides by MS/MS. Consequently, we obtained poor coverage of the primary sequence (approx. 30%; data not shown) when conducting initial peptide mapping experiments on the native (i.e. fully glycosylated) protein. Therefore, to improve coverage, and thus gain a more comprehensive view of global dynamics, HRG was enzymatically deglycosylated prior to D_2_O labelling and HDX-MS analysis. The deglycosylation protocol used enzymes that removed both N-linked and O-linked glycans. Deglycosylation was tracked by monitoring the migration of both native and deglycosylated HRG on reducing and denaturing sodium dodecyl sulphate–polyacrylamide gel electrophoresis (SDS-PAGE) (Figure 2A). To ensure that enzymatic deglycosylation did not perturb the structural integrity of HRG, we evaluated the oligomeric state of native and deglycosylated HRG by mass photometry (MP) (Figure 2B). MP enables accurate determination of the molecular weight (and thus oligomerisation state) of protein complexes under solution conditions by measuring the interference between light scattering from the protein and light reflected by the measurement surface [31]. When studied at 50 nM, both native and deglycosylated HRG were present in both monomeric and dimeric forms, with both protein species having similar oligomeric ratio. When studied at 200 nM, a mass shift towards the dimeric arrangement is observed in both species, with associated line broadening. The observed line-broadening is perhaps consistent with the behaviour of a protein containing intrinsically disordered regions (IDRs) with such regions being highly flexible and thus able to sample a greater range of open and closed conformational states [32]. Line broadening here may also reflect a dynamic equilibrium between monomeric and dimeric arrangements, as has been observed with other histidine-rich intrinsically disordered proteins [33]. As an additional control, we also studied the ability of the deglycosylated protein to undergo Zn^2+^-induced oligomerisation, a known property of the native protein [34]. The oligomerisation of native and deglycosylated HRG in the presence of zinc was studied by covalent cross-linking using bis(sulphosuccinimidyl) suberate (BS^3^) (Figure 2C). Whilst a low cross-linking efficiency likely precluded the identification of all Zn^2+^-induced multimers, a comparison of the oligomeric products mobilised on SDS-PAGE shows that Zn^2+^ enhanced the oligomerisation of deglycosylated HRG similarly to the native protein. Overall, our data indicate that the ensemble characteristics of HRG were preserved post-deglycosylation, giving us confidence in the physiological value of downstream HDX-MS analysis.

### HDX-MS reveals domain-specific dynamics

**Figure 3:**
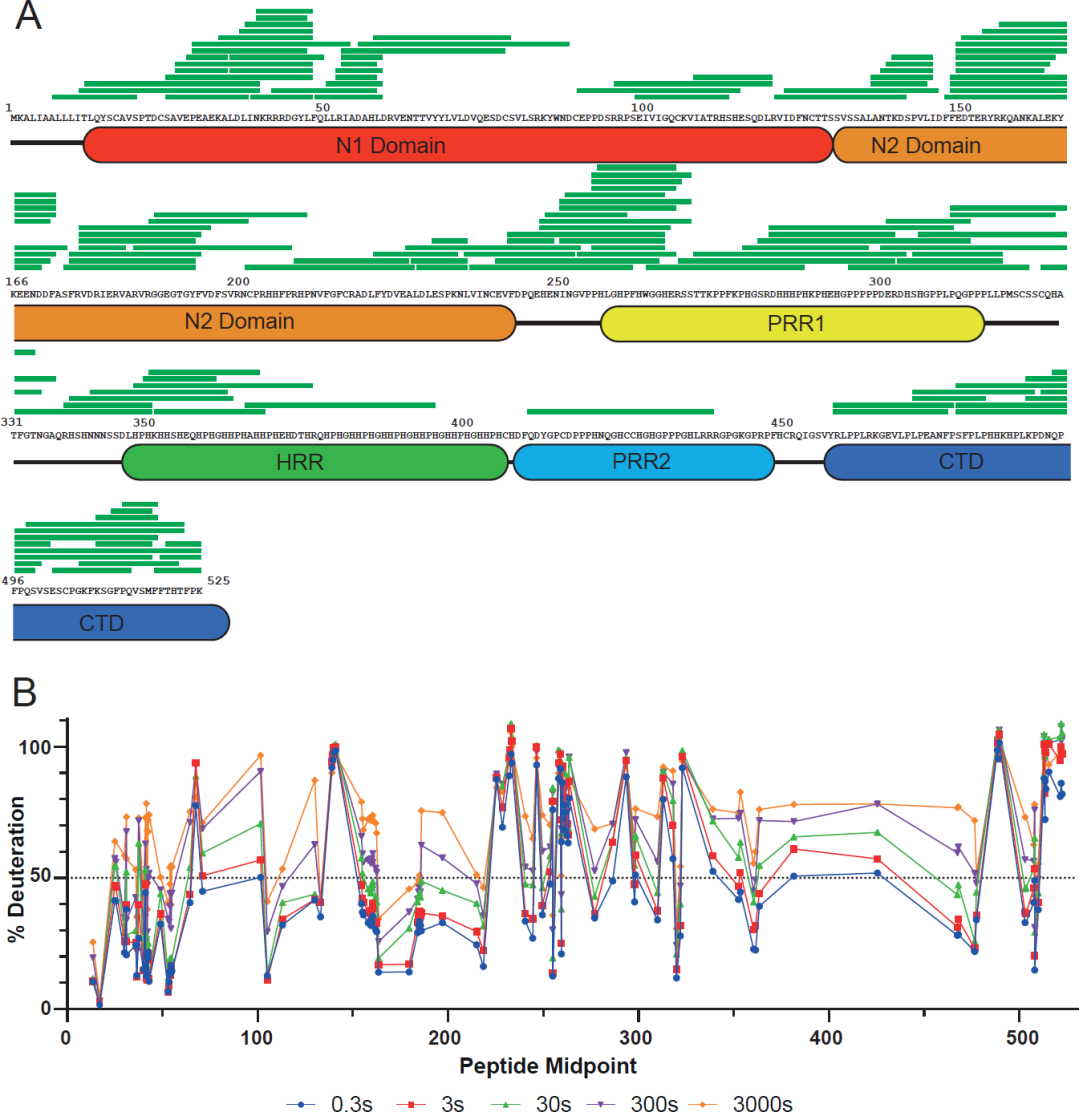
HDX-MS analysis of deglycosylated HRG. (**A**) Peptide coverage map of HRG following deglycosylation. The coverage map shows a total of 130 peptides with an average length of 18.0 amino acids. These peptides covered 92.6% of the protein with an average sequence redundancy of 4.5 peptides. (**B**) HDX-MS global uptake plot for HRG. Typically, regions where deuterium uptake is greater than 50% (shown as the dashed line) at 0.3 s incorporation are likely unstructured loops with high flexibility.

Following deglycosylation, LC-MS/MS analysis of our pepsin digests supported the assignment of 130 peptides from HRG with an overall coverage of 93% of the primary sequence (Table 1, Figure 3A). The main part of the protein that exhibited low coverage was the sequence between residues 375–455, an area consisting of part of the HRR and most of the PRR2 domains, and almost entirely made up of the pentapeptide ‘Gly-His-His-Pro-His’ motif. Lack of coverage in this region is almost certainly due to uncertainty in mapping unique peptide sequences due to the repetitive motif. Furthermore, as proline residues are essentially ‘invisible’ to HDX-MS, even if coverage of this area were improved, the resolution of the HDX-MS data would not meaningfully increase due to the high concentration of proline residues in this region. For all peptic peptides monitored, deuterium uptake followed EX2 kinetics (characterised by a gradual shift in the m/z centroid of a single isotopic envelope with incubation time [29]), and no bimodal exchange character was identified, suggesting that a single protein state had been assessed. To correct for back exchange on the UPLC system, we used a Max Deuterated control. A global deuterium uptake plot showing the deuterium uptake for each peptide at each time point is shown in Figure 3B. The raw HDX-MS data, including % deuterium incorporation for each peptide at each time point, are available in Supplementary Table S1.

**Table 1:** HDX-MS statistics for HRG experiments

Dataset/protein	Human HRG purified from human pooled plasma and enzymatically deglycosylated
HDX reaction details	50 mM Tris pH 7.4, 140 mM NaCl, 2 mM EDTA, pH 7.4; 81.7% D_2_O.
HDX time course	0.3 s, 3 s, 30 s, 300 s, 3000 s
HDX controls	Non-deuterated, maximally deuterated
Number of peptides	130
Sequence coverage	92.6%
Average peptide length/redundancy	18.0 amino acids, 4.5 redundancy
Replicates (biological or technical)	4 non-deuterated, 3 deuterated, 3 maximally deuterated All used the same protein stock, exchange reactions were independent
Repeatability	Mean standard deviation for three repeats = 1.4%

### Structural insights from HDX-MS and AlphaFold integration

**Figure 4:**
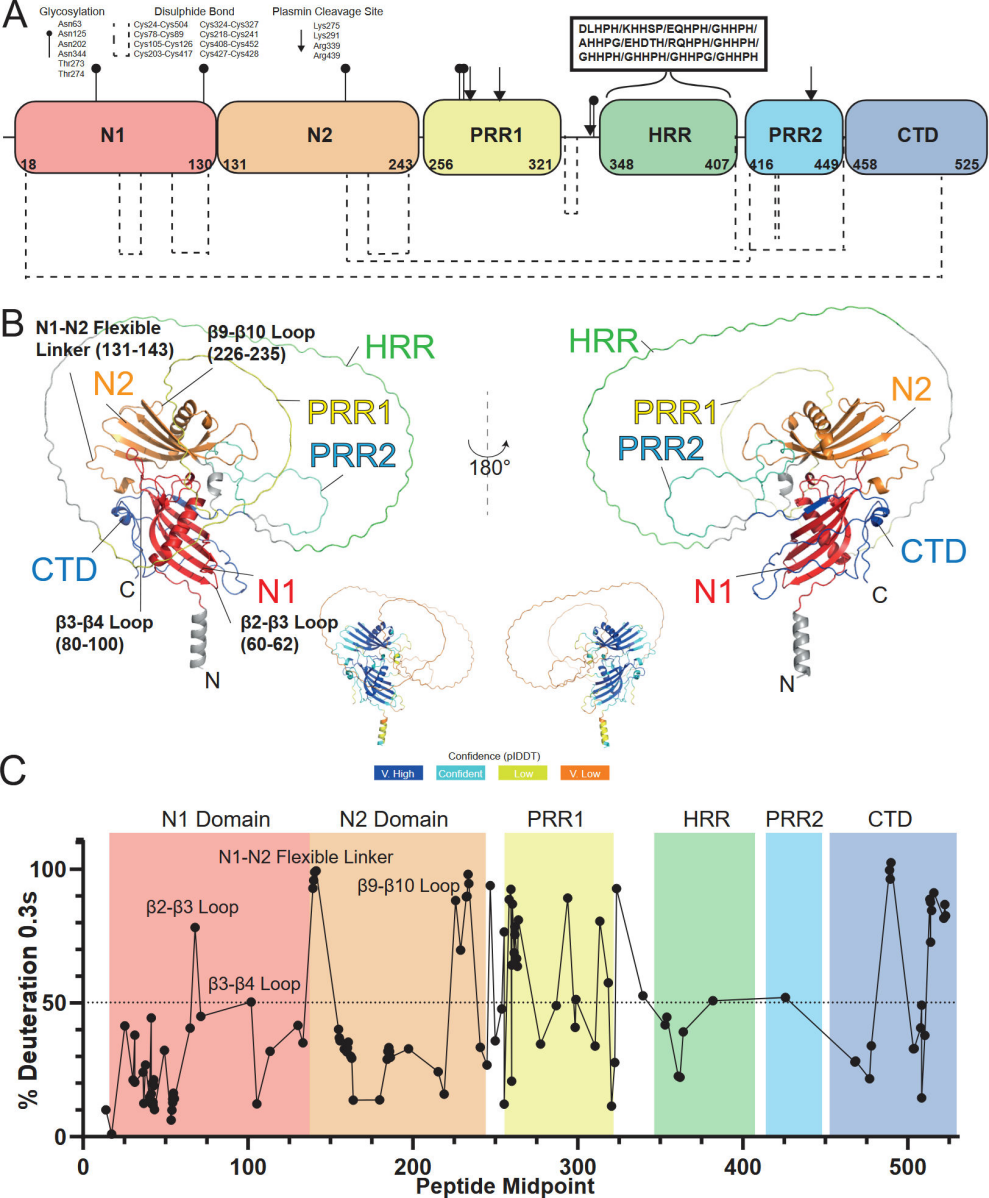
Predicted model of HRG generated by AlphaFold and comparison to HDX-MS data. (**A**) Domain structure of HRG; HRG contains a tandem cystatin-like fold (**N1 and N2**), two proline-rich regions (PRRs) which flank a histidine-rich region (HRR) containing the conserved pentapeptide motif ‘GHHPH’, followed by a C-terminal domain. Note that HRG was deglycosylated prior to D_2_O labelling. (**B**) Views of the HRG 3D-structure acquired from the AlphaFold database (AF-P04196-F1-v4). AlphaFold produces a per-residue confidence score (predicted local distance difference test, pLDDT) between 0 and 100. Some regions with a pLDDT score below 50 may be unstructured in isolation. Model confidence is indicated as follows; dark blue; very high confidence (pLDDT > 90), light blue; confident (90 > pLDDT > 70), yellow; low confidence (70 > pLDDT > 50) and orange; very low confidence (pLDDT < 50). (**C**) HDX-MS data for HRG at 0.3 s of deuterium incubation. Typically, regions where deuterium uptake is greater than 50% (shown as the dashed line) at 0.3 s incorporation are likely unstructured loops with high flexibility.

**Figure 5:**
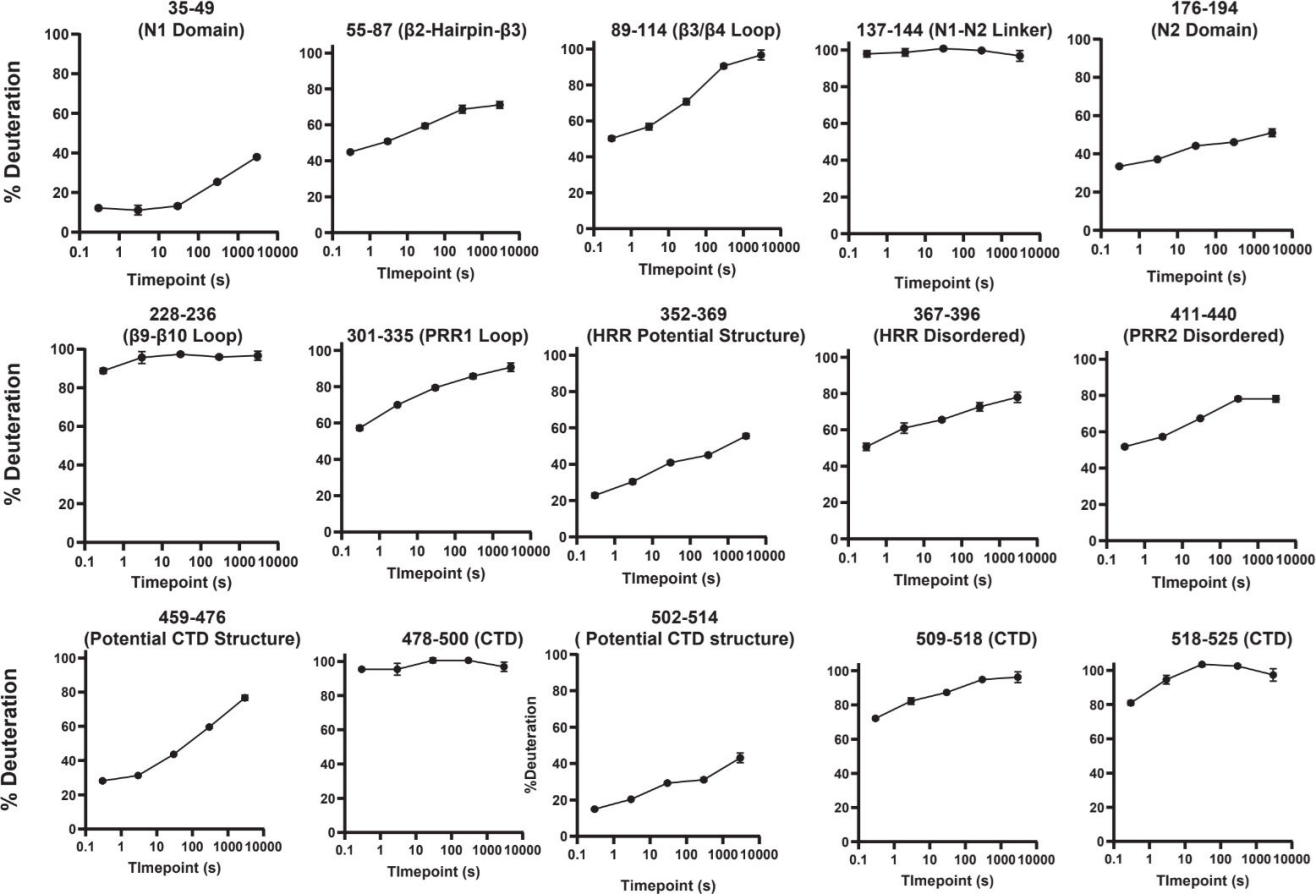
Representative D_2_O uptake plots for HRG peptides displaying fast and slow solvent exchange. Peptides shown are representative of regions of HRG that showed either high solvent exchange (likely corresponding to either disordered stretches or highly dynamic regions of secondary structure) or low solvent exchange (likely corresponding to regular secondary structure and/or interdomain interactions). Error bars represent one standard deviation from three independent D_2_O exchange reactions (n=3) - some error bars are smaller than the points depicted. (Note, deuterium uptake plots of all peptides of HRG are also shown in Supplementary Figure S3)

To aid interpretation of our HDX-MS data, the domain structure of HRG is provided (Figure 4A). Additionally, to provide a better three-dimensional context for these HDX-MS data, we have utilised the AlphaFold model of human HRG (AF-P04196-F1-v4) (Figure 4B) [22]. Note, when referring to individual secondary structure elements in this model, we have adopted the nomenclature established for the solved structure of fetuin-B [35] (PDB: 6HPV), a paralog with a similar tandem cystatin-type domain architecture. Most residues in the N1 and N2 domains exhibited slow solvent exchange, which is consistent with a compact fold for these domains. However, several regions within N1/N2 displayed rapid exchange (where the peptide was >50% deuterated at the 0.3 second incubation), providing evidence for more dynamic stretches of secondary structure and/or flexible loops within these regions (Figure 4C). Representative peptide uptake plots for both fast and slow-exchanging regions are shown in Figure 5. Within N1, residues 57–78 (corresponding to the end of β2, a short hairpin turn and most of β3) exhibited fast exchange and may therefore indicate a dynamic stretch of secondary structure within the core protein. Similarly, residues 89–114 (corresponding to an extended loop between β3–β4 and most of β4) were also fast exchanging. Notably, residues 135–144, positioned between N1 and N2, showed high exchange, consistent with a flexible linker between these domains. In N2, residues 222–236, corresponding to a loop connecting β9–β10, also showed high H/D exchange rates.

Residues 242–458, incorporating PRR1, HRR and PRR2 domains, broadly showed high rates of H/D exchange indicating these regions are solvent exposed and, considering the physical properties of the amino acids comprising this stretch (i.e. low sequence complexity, high net charge and low hydrophobicity and no amide hydrogen for participation in hydrogen bonding for PRR1/PRR2), may be largely disordered *in vivo*. These findings are supported by disorder predictions (Supplementary Figure S1), the low AlphaFold confidence score (average pLDDT = 37.46), and high positional uncertainty of these residues as indicated in the AlphaFold-predicted alignment error (PAE) map (Supplementary Figure S2). While large sections of the PRR1/HRR/PRR2 stretch demonstrated high solvent exchange consistent with predominantly disordered character, there was some evidence for short stretches of lower exchanging residues. However, the relatively long length of peptic peptides in this stretchprecludes specific interrogation. An area with unambiguous low solvent exchange included residues 352–369, corresponding to the start of the HRR. This is unlikely to reflect regular secondary structure and may have topological origins.

Residues 459–482, incorporating the start of the CTD, exhibited a relatively slow solvent exchange rate which may indicate the formation of some stable secondary structure and/or the presence of local interactions in this area. The AlphaFold model predicts the initial part of the CTD chain adopts a beta strand structure where residues 457–465 run parallel to strand β5 of the β-sheet involving N1. Inspection of the AlphaFold PAE plot indicates high confidence in the relative positions of N1/N2 and the CTD. The Encyclopaedia of Domains (TED) also assigns N1 and the CTD to the same structural superfamily (CATH 3.10.450.10, boundaries; 19–139, 455–519, Qscore; 73.68) suggesting that these regions, while discontinuous in sequence, act as a single functional unit (Supplementary Figure S2). Given these observations, the slow exchanging residues observed at the start of the CTD may resemble a compact region featuring interactions between the CTD and N1. Residues 478–501 showed a high level of exchange indicating disorder in this stretch. Residues 502–514, however, exhibited relatively slow solvent exchange and may resemble some ordered structure and/or local interactions in this region (potentially stabilised by the disulphide bond at Cys24-Cys504). Residues 509–525 again exhibited fast exchange rates. Inspection of the AlphaFold model indicates that the remaining 480–525 residues of the CTD lack regular secondary structure and appear to run irregularly along the surface of the N1 domain. It is therefore likely that the remaining residues of the CTD are largely disordered.

## Discussion

In the present study, we used HDX-MS to gain insights into the structure and dynamics of intact HRG in solution. Such investigations are highly relevant given that HRG poses significant challenges for traditional high-resolution structural methods. For instance, its moderate molecular weight (~75 kDa) presents a challenge for in-solution NMR, while the flexibility and heterogeneous glycosylation impair crystallisation, which make the whole-length protein a challenging target for X-ray crystallography. Similarly, the combination of small size, flexibility and glycan content severely limits the resolving power of single-particle cryo-EM. As such, HRG lies in a methodological blind spot for classical structure determination. Notably, the HDX-MS workflow can provide in-solution structural information for proteins that are recalcitrant to classical structure determination. While previous biophysical data on HRG exists, such work has primarily been conducted on either synthetic peptides or fragments derived from proteolytic cleavage of the native protein. Information gained by HDX-MS provides information on the regional dynamics of the whole-length protein, thus factoring in dynamical contributions arising from domain topology. Our study is therefore able to provide a more holistic representation of the whole-length HRG structure. Moreover, where previous biophysical approaches – such as CD on proteolytic fragments – can only provide information at the domain level, HDX-MS provides information at the peptide level, making this approach more informative.

The findings provide evidence that the N1 and N2 domains exhibited predominantly slow-exchanging profiles, consistent with a compact globular fold. Notably, the structure of the N2 domain of rabbit HRG has previously been found to possess a characteristic cystatin-like fold [6]. The AlphaFold model predicted a tandem cystatin-like arrangement with high confidence (average pLDDT = 85.97). Comparing exchange data to the AlphaFold model, we found good agreement between slow deuterium uptake and the likely folded sections. Areas of high exchange that correlated well to likely solvent-exposed hairpin turns connecting individual β-strands, but also extended stretches predicted to form flexible loops were also observed. This included two likely flexible loops connecting β3–β4 on N1, and another loop connecting β9–β10 on N2. Flexible loop regions are known to play a critical role in the biological function of many proteins and have been shown to be involved in ligand binding. It is possible that these regions act as flexible epitopes mediating interactions between HRG and other proteins. Another area of high exchange correlated well to the exposed linker joining N1 and N2. This linker is likely to be necessary to allow independent folding of the N1 and N2 domains during ribosomal translation, as well as acting as a hinge allowing these domains to move relative to one another. It is apparent that the acquisition of different N1/N2 domain-domain orientations mediated by this linker may permit efficient binding of HRG to a range of different protein targets, thus contributing to its multifunctionality. The N-terminal domains of HRG are also homologous to the heparin-binding region of antithrombin III, where a positively charged helix mediates interaction with heparin [6,19,36]. Similar helical regions are involved in glycosaminoglycan binding in other proteins, such as protease nexin-1, whose dimeric crystal structure reveals heparin binding at the interface between two helices (one from each monomer) via key lysine residues [37]. HRG is known to neutralise heparin and may do so via a comparable mechanism. Specifically, helices from the N1 and N2 domains (which contain lysine residues) could together form a bivalent heparin-binding site.

The data also revealed a high degree of H/D exchange within the PRR1/HRR/PRR2 stretch. It is likely that these regions do not adopt compact secondary structure and are exposed to the aqueous environment on the protein surface. This is in general agreement with previous biophysical investigations on isolated HRG domains. Notably, Borza et al*.* probed the secondary structural characteristics of the isolated HRR-PRR using CD spectroscopy, which indicated a high poly-proline-II helix content [2]. A separate study by Martin et al. utilised CD and NMR to study the secondary structure of a synthetic peptide corresponding to the HRR-PRR2 region, with both techniques indicating a largely disordered structure [38]. The PRR1/HRR/PRR2 stretch also exhibited a low average pLDDT score which has previously been shown to correlate with protein disorder [22]. The flexible nature of the HRR is likely to be important for its functional roles, including the binding of Zn^2+^ ions, a known HRG cofactor [19,39–42]. Modelling studies predict that Zn^2+^-co-ordinating histidine residues project outward from the extended HRR, allowing efficient chelation of metal ions [2]. We show that Zn^2+^-binding is important for oligomerisation of HRG, consistent with previous data [34]. It is possible that oligomerisation may be driven, at least in part, by zinc bridging histidine residues on the HRRs of adjacent HRG molecules, akin to the proposed mechanism of oligomerisation of the histidine-rich histatin proteins [33]. However, there is broad evidence to suggest Zn^2+^ binding at the HRR drives multiple structural changes across the entire HRG molecule (Supplementary Table S2) [14,19,34,38,43,44], which may additionally facilitate oligomer formation. Co-ordination of Zn^2+^ at the HRR is shown to enhance the binding of HRG to cell surface heparin sulphate via the N1/N2 domains, suggesting Zn^2+^-binding at the HRR drives structural changes in these domains, which may promote multimer formation [19]. Indeed, it is noted that the structure of the N2 domain was solved as a dimeric arrangement, suggesting a potential role of this domain in multimer formation [6].

We found that residues 459–480 of the CTD were relatively slow to exchange, which may reflect some ordered structure and/or local interactions in these regions. Inter-domain contacts between the CTD and the HRR (potentially stabilised by the Cys408-Cys452 disulphide) may explain the lower solvent exchange here. Inspection of the AlphaFold model also shows that the 457–465 N-terminal residues of the CTD form a β-strand that extends the β-sheet in the N1 domain. Insights from both AlphaFold and the CATH domain database indicate the N1 and CTD domains may be part of a single functional unit. The N1/N2 domains and the CTD have typically been studied as individual domains when ascertaining their structural and functional properties. It is apparent that the contribution of the CTD to the overall N1 fold may introduce new properties that are not seen when studying these regions in isolation, and this requires further investigation. Our data revealed a high degree of H/D exchange within the remaining residues of the CTD (509-525), indicating that these residues are likely solvent exposed and may lack regular secondary structure, which is again in good agreement with the AlphaFold model. A high level of disordered content in this region is also supported by CD spectroscopic studies on the isolated CTD by Borza *et al*., which revealed significant random coil character and pronounced susceptibility to proteolytic cleavage in this region [2]. Previous work by Jones *et al*. has suggested lysine residues within the CTD of HRG are important for plasminogen binding, although the targeted removal of the C-terminal lysine on HRG did not perturb its ability to bind plasminogen [20]. This largely supports our HDX-MS data where it is likely that the structural plasticity of the CTD means there is no single preferred lysine binding residue for plasminogen. The CTD shares homology with the CLESH-1 motif in CD36, which mediates binding to thrombospondin type 1 repeat (TSR) domains in TSP-1, TSP-2 and vasculostatin [45–47]. Modelling by Klenotic et al. showed the CD36 CLESH motif binds TSRs via a flexible, extended conformation [48]. NMR titrations confirmed the CLESH domain remains disordered upon binding TSR2, supporting a ‘fuzzy’ interaction mechanism driven by an ensemble of conformations rather than rigid residue-specific contacts [48]. Given the presence of a homologous motif within the CTD of HRG, and its similar disordered character, these findings are likely to be pertinent to HRG. The largely unstructured nature of the CTD may enable HRG to interact dynamically with multiple binding partners, including TSP-1, TSP-2 and vasculostatin.

Recent findings by Lv et al. have demonstrated that three of the disulphide bonds located in proximity to the HRR (namely, Cys324-Cys327, Cys408-Cys452 and Cys427-Cys428) act as substrates for protein disulphide isomerase (PDI) [4]. Reduction of these bonds by PDI has been shown to enhance the interaction of HRG with both heparin and activated factor 12 (FXIIa) - both of which have been shown to bind at the HRR [49,50]. Thus, certain binding sites within the HRR may be cryptic and the accessibility of these sites to their binding partners appears to be, to a certain extent, regulated by these allosteric disulphide bonds. Whilst HDX-MS does not have sufficient resolution to comment on the dynamics of individual amino acids, based on the high degree of solvent exchange observed within the PRR1/HRR/PRR2 stretch, it is likely these disulphide bonds are largely solvent exposed thus providing access for their reduction by redox enzymes *in vivo*. Reduction of the Cys203-Cys417 and Cys408-Cys452 disulphide bonds, in combination with plasmin cleavage, is needed to liberate the functional HRR peptide from the intact protein. Notably, all putative plasmin cleavage sites are also located within the exposed PRR1/HRR/PRR2 stretch and are thus likely to be susceptible to proteolysis. However, it is also apparent that native HRG protein would have mechanisms to limit premature proteolysis under basal conditions. HRG has been shown to bind plasminogen with micromolar affinity and may be a potential plasma carrier of plasminogen *in vivo* [17]. It is conceivable that the complexation of HRG with plasminogen limits accessibility of the HRR/PRR stretch, thus controlling unchecked proteolysis. Subsequent cleavage of plasminogen by tPA may induce a conformation change in the plasmin molecule that facilitates proteolysis of HRG, indicating a bidirectional interaction between plasmin(ogen) and HRG. Such a mechanism would be analogous to the proposed mechanism of regulation of paralogous high molecular weight kininogen (HK), which circulates in complex with plasma kallikrein (PK) and factor XI (FXI) [51]. Proteolytic activation of PK by factor XIIa (FXIIa) reorients its protease domain, enabling activated PK to cleave HK and release both bradykinin and activated HK (HKa) [51].

This study has several limitations, mostly arising from the biological complexity of HRG. For instance, since we did not have the ability to detect glycopeptides via MS/MS, the HRG protein was deglycosylated prior to HDX-MS measurements. Thus, the study cannot claim to measure HRG structure and dynamics under truly native conditions. Whilst glycan chains are unlikely to contribute significantly to local or global protein structure (as has been robustly demonstrated by [52]), they may influence protein dynamics. Indeed, there is broad evidence in the literature to suggest the attachment of bulky glycan chains enhances protein thermodynamic stability by restricting the flexibility of the polypeptide chain [52,53]. Molecular dynamics studies suggest greater overall thermodynamic stability of the glycosylated protein is afforded through glycan-mediated destabilisation of the unfolded state, where glycan chains prohibit the unfolded protein from forming residual structure, instead forcing the chain to adopt an energetically unfavourable extended conformation [53]. Thus, it is possible our study may overstate the dynamics of HRG within certain regions, especially in areas located near to the glycosylation sites. Future studies incorporating in-line deglycosylation after deuterium labelling would certainly be valuable, although some caution is advised. HRG from pooled plasma has been shown to exhibit some glycoheterogeneity [5], such that maintaining its glycosylation profile during deuterium exchange could produce mixed exchange patterns arising from differently glycosylated variants. Additionally, the required additional time to allow efficient deglycosylation may lead to an increase in back exchange, causing deuterium signal loss and thus reducing spatial resolution—a key challenge in HDX-MS analysis of heavily glycosylated proteins. Unfavourable back-exchange kinetics may be especially prohibitive in the case of HRG where the large number of polar side chains in the HRR and PRR regions are unlikely to retain deuterons for the time periods required for sufficient deglycosylation [54]. Thus, deglycosylation prior to D_2_O labelling ultimately remains advantageous, as it avoids complications arising from glycoheterogeneity and ensures good coverage and clearer interpretation of deuterium exchange data. Finally, it is noted that further insights from our HDX-MS approach could be revealed by performing D_2_O labelling reactions at different temperatures (temperature-dependent-HDX-MS). This would allow for a better differentiation of dynamics in certain regions. This iteration of the HDX-MS technique has also been successfully applied to other coagulation proteins [55].

In conclusion, this study provides the first comprehensive assessment of the conformational landscape and intrinsic dynamism of full-length HRG under near-native conditions. By integrating experimental HDX-MS data with computational predictions, we establish a structural framework that reveals extensive disorder and flexibility across key functional domains. This architectural insight provides a mechanistic basis for HRG’s remarkable functional diversity, particularly in the regulation of coagulation, fibrinolysis and immune responses. Beyond advancing our understanding of HRG’s inter-domain organisation, these findings open new avenues for exploring its molecular interactions and targeting its activity in therapeutic contexts. As such, this work lays a critical foundation for future investigations into the structural determinants of HRG’s biological roles and its potential as a modulator in complex physiological and pathological processes. Finally, it is worth noting that while HDX-MS has typically been applied to recombinant proteins purified from heterologous expression systems, this work demonstrates the ability of the HDX-MS workflow to yield structural insights into proteins extracted directly from complex biological matrices.

## Materials and methods

### Disorder predictions

The human HRG sequence was obtained from the Uniprot server (Uniprot ID: P04196). Disorder predictions were obtained using the following predictors: IUPRED3A_long [23], IUPred3A_short [23], PrDOS [24], VLXT [25], VL3 [26], VSL2 [27] and ESpritzX [28] (accessed 10/06/2025). The raw data were extracted and visualised using GraphPad (Prism).

### Purification of HRG from human plasma

HRG was purified from human citrated pooled plasma using nickel-NTA affinity chromatography. Plasma aliquots (50 ml) were thawed in a water bath at 37°C. During thawing, aliquots were supplemented with an ethylenediaminetetraacetic acid (EDTA)-free protease inhibitor cocktail tablet (Roche). Additionally, aprotinin and ε-aminocaproic acid (Sigma) were added to a final concentration of 50 kallikrein inhibitory units and 40 mM respectively. Thawed plasma was vacuum filtered using a 0.45 µm filter. Imidazole was then added to a final concentration of 20 mM. The sample was then loaded onto a HisTrap HP nickel-nitrilotriacetic acid column (Cytiva) operated by an ÄKTA pure^TM^ FPLC system. The column was pre-equilibrated with 5 column volumes (CVs) of 50 mM Tris-HCl, 1 M NaCl, 5% glycerol, 20 mM imidazole, pH 7.4. After sample binding, the column was washed with 50 mM Tris-HCl, 1 M NaCl, 5% glycerol, 200 mM imidazole, pH 7.4 until a stable UV reading was reached (i.e., where UV did not fluctuate > 0.1 mAU for >1 min; this was approximately 20 CVs). HRG was eluted by washing the column with 5 column volumes of 50 mM Tris-HCl, 1 M NaCl, 5% glycerol, 400 mM imidazole, pH 7.4. Fractions corresponding to HRG were collected and concentrated using a Vivaspin filter with a molecular weight cutoff of 50 kDa (Sartorius, Epsom, UK). The purified protein was then buffer-exchanged in 50 mM Tris-HCl, 140 mM NaCl, pH 7.4. The purity of the protein was examined by SDS-PAGE under reducing and denaturing conditions. The concentration of the purified HRG protein was determined using the bicinchoninic acid (BCA) assay. HRG aliquots were stored at −20°C until analysis.

### CD spectroscopic analysis of HRG structure

Far UV CD spectra of HRG were obtained using a mos-500 spectropolarimeter (Bio-Logic). Far UV CD spectroscopy measurements used 0.2 mg/ml of HRG. HRG was prepared in 50 mM Tris-HCl, 140 mM NaCl buffer, pH 7.4. CD spectroscopy measurements were conducted at 20°C. The cell pathlength was 0.1 cm. Scans were conducted over 0.5 nm intervals with an acquisition time of 0.5 s and bandwidth of 2 nm. Scans were performed in triplicate and averaged. Spectra were corrected by subtracting a protein-free buffer blank. To provide an estimate of secondary structure content, the CD spectrum was deconvoluted using the BeStSel algorithm [30].

### HRG deglycosylation

HRG was deglycosylated under native conditions using the NEB protein deglycosylation mix II (New England Biolabs). The mixture contained peptide:N-glycosidase F (glycerol-free), O-glycosidase, α2–3,6,8,9 neuraminidase A, β1–4 galactosidase S and β-N-acetylhexosaminidase. This mix is designed to simultaneously remove both N-glycans and some O-glycans. Briefly, 2 mg/ml of HRG in 50 mM Tris-HCl, 140 mM NaCl, pH 7.4 was incubated with 5 µl of 10 × protein deglycosylation mix II and 5 µl of 10 × deglycosylation buffer I. The final reaction volume was 50 µl. HRG was deglycosylated for 2 hours at 37°C. The extent of deglycosylation was assessed using mobility shift in SDS-PAGE under reducing and denaturing conditions.

### Mass photometric analysis of glycosylated and deglycosylated HRG

Solution-phase mass determination of native and deglycosylated HRG was performed using the TwoMP (Refeyn) mass photometer. Native and deglycosylated HRG samples were prepared at either 50 nM or 200 nM in 50 mM Tris-HCl, 140 mM NaCl, 2 mM EDTA, pH 7.4. Experimental data were collected using High Precision glass microscope slides, repeatedly cleaned in ultrapure water and isopropanol and dried using a stream of nitrogen gas. Data were obtained through the collection of mass photometry videos recorded for one minute using the AcquireMP v2.5 software (Refeyn). Prior to data collection, mass calibration was conducted using bovine serum albumin (66 kDa) and aldolase (160 kDa). By fitting Gaussian functions to ratiometric contrast values obtained from the protein standards using the DiscoverMP v2.5 software (Refeyn), a linear mass calibration was obtained. The experimental data were then fitted to this calibration, and graphs were generated using the DiscoverMP v2.5 software (Refeyn).

### Chemical cross-linking to assess HRG oligomerisation in presence of Zn^2+^

The oligomeric properties of native and deglycosylated HRG at different Zn^2+^ concentrations were investigated via chemical cross-linking. Briefly, HRG at 5 µM in 20 mM PBS, pH 7.4 was incubated with either 0, 5, 25, 50, 100 or 250 µM ZnCl_2_. To each reaction, BS^3^ (ThermoFisher Scientific) was added to a final concentration of 1 mM. The final reaction volume was 30 µl. The cross-linking reactions were allowed to proceed for 1 h at room temperature. Cross-linking reactions were quenched via the addition of 1 M Tris-HCl, pH 7.4. Cross-linked products were identified by Coomassie-stained SDS-PAGE under reducing and denaturing conditions.

### HDX-MS analysis of deglycosylated HRG

HRG was deglycosylated prior to D_2_O labelling and HDX-MS analysis (see above). Exchange reactions of deglycosylated HRG in deuterated buffer were performed manually. In brief, 5 μl of 5 μM deglycosylated HRG in protein dilution buffer (PDB; 50 mM Tris-HCl, 140 mM NaCl, 2 mM EDTA, pH 7.4) was incubated with 45 μl of D_2_O buffer (50 mM Tris-HCl, 140 mM NaCl, 2 mM EDTA, 90.8% D_2_O; pH 7.4). The D_2_O exchange reactions were performed at 5 time points: 0.3 s, 3 s, 30 s, 300 s and 3,000 s (with the 0.3 s time point being a 3 s time point conducted at 0°C). For each time point, D_2_O exchange reactions were carried out in triplicate. At the end of their respective incubations, D_2_O exchange reactions were quenched by the addition of 20 μl ice-cold quench buffer (2 M urea and 2% formic acid) to a final pH of approx. 2.5. After the addition of quench buffer, samples were immediately snap-frozen in liquid nitrogen and stored at −80°C until analysis. A maximally deuterated control was also produced by incubating 0.5 μl of 50 μM deglycosylated HRG with 64.5 μl of deuterated quench solution (final D_2_O concentration 97.24%) for 48 h.

Quenched samples were thawed at room temperature and 50 μl of each sample was injected onto an Acquity UPLC M-Class System with HDX Technology. The quenched samples were passed through an immobilised Enzymate Pepsin column (Waters, 186007233) at a flow rate of 200 μl/min at 15°C. The resulting peptic peptides were then transferred onto an Acquity UPLC Peptide BEH C18 VanGuard Pre-column for 2 min at 0.1°C. Peptides were separated using a linear acetonitrile gradient (5–35%) over 7 min at a flow rate of 40 μl/min. Mass spectra were acquired on a Waters Select Series cyclicIMS mass spectrometer with an electrospray ionisation source, with a single pass around the cyclic IMS, with a spray voltage of 3.0 kV, and an m/z range of 50 to 2000. The data were collected in HDMSe mode. Peptic peptides were identified using non-deuterated control samples which were prepared in parallel to the deuterated samples. Four replicates were used to identify non-deuterated peptides.

Non-deuterated peptides were identified using the Protein Lynx Global Server software (PLGS, Waters). Peptide filtering and deuterium incorporation were performed using HDExaminer (Sierra Analytics). The following criteria were used to include peptides in the HDX-MS dataset: minimum intensity of 5,000; minimum sequence length of 5; maximum sequence length of 25; a minimum of three fragment ions; a minimum of 0.1 products per amino acid; a minimum score of 5.00; a maximum MH^+^ error of 5 ppm.

## Supplementary material

Online supplementary material 1

## Data Availability

The mass spectrometry data have been deposited to the ProteomeXchange Consortium via the PRIDE partner repository [56], with the dataset identifier PXD065786.

## Open Access

This article has been published open access under our Subscribe to Open programme, made possible through the support of our subscribing institutions, learn more here: https://portlandpress.com/pages/open_access_options_and_prices#conditional

## Abbreviations

BCA: bicinchoninic acid
BS^3^: bis[sulphosuccinimidyl] suberate
CD: circular dichroism
CID: collision-induced dissociation
CLESH-1, CD36, LIMP2: EMP structural homology
CTD: C-terminal domain
EDTA: ethylenediaminetetraacetic acid
ETD: electron transfer dissociation
HDX-MS: hydrogen-deuterium exchange mass spectrometry
HK: high molecular weight kininogen
HRG: histidine-rich glycoprotein
HRR: histidine-rich region
LC-MS/MS: liquid chromatography-tandem mass spectrometry
PAE: predicted alignment error
PRR: proline-rich region
SDS-PAGE: sodium dodecyl sulphate-polyacrylamide gel electrophoresis
TSR: thrombospondin type 1 repeat
UV: ultraviolet
pLDDT: predicted local distance difference test
tPA: tissue plasminogen activator
