# Supplementary material for: Investigating the conformational landscape of human histidine-rich glycoprotein using amide HDX-MS
Source: Biochem J. 2025 Nov 17;482(22):1721–35. doi: 10.1042/BCJ20253366 (PMC12751054; doi:10.1042/BCJ20253366)
Supplement: Online supplementary material 1 [file bcj-482-22-BCJ20253366-s001.pdf]

Supplementary Information

Investigating the conformational landscape of human histidine-rich glycoprotein using amide HDX-MS

Stephen J. Hierons, Boyang Lin, Remi Fritzen, Claudia A. Blindauer, Ramzi A. Ajjan, Glenn R. Masson, and Alan J. Stewart

Table S1. Raw HDX-MS data, including % deuterium incorporation for each peptide at each time point.

Table S2. Observed structural adaptations of HRG in the presence of Zn^{2+}.

Figure S1. HRG predicted disorder using an eclectic range of computational predictors.

Figure S2. AlphaFold structural model for human HRG showing predicted alignment error (PAE) plot and The Encyclopaedia of Domains assignment.

Figure S3. D_2O uptake plots for all HRG peptides.

Table S1. Raw HDX-MS data, including % deuterium incorporation for each peptide at each time point. The start and end numbers refer to the position of the first and last amino acids in each peptide, respectively.

Protein	Start	End	Apo - 0.3s	Apo - 3s	Apo - 30s	Apo - 300s	Apo - 3000s
P04196	7	20	10.54	10.61	11.66	19.61	25.49
P04196	11	39	41.41	46.95	54.59	57.43	63.83
P04196	12	22	1.56	3	3.34	2.83	4.06
P04196	12	39	41.13	46.48	54.3	56.04	63.68
P04196	25	37	38.01	39.71	52.17	67.56	73.2
P04196	25	47	24.35	25.31	29.98	42.51	53.19
P04196	26	34	21.46	25.92	29.1	34.92	58.56
P04196	26	47	12.9	12.31	13.27	22	35.21
P04196	28	34	20.71	25.7	28.11	33.88	57.28
P04196	29	46	26.95	39.65	63.2	71.75	72.81
P04196	29	53	44.33	47.53	53.16	62.92	72.88
P04196	33	47	15.14	14.6	16.07	29.51	42.7
P04196	35	47	16.37	15.76	17.99	34.87	50.88
P04196	35	49	12.24	11.13	13.21	25.35	38
P04196	36	47	12.64	11.72	14.31	25.54	37.62
P04196	37	47	20.46	19.58	23.25	47.37	67.77
P04196	38	45	20.22	20.73	28.41	53.19	78.31
P04196	38	46	13.3	13.66	15.51	29.38	47.87
P04196	38	47	21.57	21.48	25.2	49.85	72.02
P04196	39	46	19.39	18.64	23.12	49.47	67.6
P04196	39	47	10.6	11.73	12.48	51.72	73.99
P04196	41	57	32.38	36.26	43.93	45.4	50.25
P04196	48	58	6.75	6.35	12.23	34.25	40.68
P04196	49	58	10.42	8.97	15.19	33.25	39.82
P04196	50	58	13.14	12.83	17.62	39.08	47.59
P04196	51	57	14.41	13.85	19.15	43.93	53.93
P04196	51	58	14.18	14.61	19.6	43.91	54.64
P04196	52	57	16.67	16.55	18.27	30.65	39.78
P04196	52	58	14.66	14.14	19.31	44.19	54.13
P04196	52	77	40.56	43.67	53.92	71.05	75.23
P04196	55	87	44.84	50.86	59.42	68.67	71.1
P04196	57	78	77.57	93.75	88.75	92.79	80.85
P04196	89	114	50.19	56.77	70.66	90.5	96.64
P04196	98	112	12.72	11.11	13.86	29.51	41.03
P04196	107	119	32.07	34.37	40.6	46.72	53.44
P04196	120	140	41.54	41.77	43.74	62.65	87.12
P04196	121	145	35.11	40.71	41.3	40.44	40.67
P04196	135	143	91.97	94.24	94.91	93.08	89.98
P04196	135	144	94.94	96.83	97.74	96.52	94.12
P04196	136	144	97.9	99.66	100.4	99.14	96.86
P04196	137	144	97.87	98.64	100.47	99.8	96.71

P04196	138	144	98.39	99.74	100.86	99.35	96.99
P04196	146	170	32.82	36.82	45.75	56.61	72.24
P04196	147	171	33.22	37.16	46.2	57.08	73.01
P04196	147	172	31.86	36.3	44.23	55.54	71.83
P04196	147	174	33.13	38.19	47.35	55.96	71.78
P04196	148	161	40.09	47.21	57.63	65.74	78.98
P04196	148	162	36.95	42.11	51.72	59.17	72.43
P04196	148	163	35.91	40.7	48.88	55.98	68.23
P04196	148	171	34.07	37.44	44.55	55.87	73.14
P04196	148	172	33.33	37.29	46.33	57.36	73.19
P04196	149	172	35.4	40.23	48.63	59.3	73.86
P04196	152	172	30.16	33.35	42.85	53.66	70.77
P04196	153	172	29.46	32.89	40.89	52.12	67.08
P04196	155	172	14.03	16.92	19.4	25.69	34.46
P04196	174	194	29.07	33.15	40.97	41.93	47.53
P04196	175	194	32.05	35.74	42.75	44.65	49.16
P04196	175	195	29.78	34.67	42.91	44.39	48.86
P04196	176	183	14.13	17.12	30.8	37	45.76
P04196	176	194	33.44	37.28	44.25	46.28	50.99
P04196	176	195	31.9	35.63	42.65	44.68	49.12
P04196	176	196	29.8	36.59	48.68	62.39	75.6
P04196	185	209	32.97	35.44	45.27	57.65	74.81
P04196	202	228	24.49	29.57	40.31	47.84	51.23
P04196	210	227	16.26	22.28	31.68	35.48	46.37
P04196	222	235	69.24	76.86	84.89	85.65	82.89
P04196	223	228	87.46	88.13	88.53	89.49	84.35
P04196	227	254	33.54	36.43	47.63	54.17	73.43
P04196	228	236	88.85	95.73	97.39	95.99	93.66
P04196	229	236	89.09	98.79	98.25	96.61	94.77
P04196	230	236	97.12	106.91	108.74	106.38	105.49
P04196	231	236	93.74	102.02	103.75	102.37	100.02
P04196	236	253	27	34.44	47.39	52.56	64.94
P04196	237	262	35.85	39.42	46.22	60.09	73.85
P04196	241	266	47.58	52.35	58.32	61.75	70.18
P04196	243	250	93.02	99.82	100.42	97.93	95.65
P04196	243	267	75.93	79.25	84.27	82.58	83.13
P04196	248	268	87.82	93.73	98.73	92.09	89.77
P04196	248	271	21.05	25.13	38.07	43.7	50.79
P04196	249	261	12.64	13.66	19.5	30.21	35.69
P04196	251	267	91.6	97.19	98.14	96.13	93.67
P04196	251	269	86.19	92.43	93.43	90.44	89.24
P04196	251	271	68.2	73.18	72.71	73.21	70.21
P04196	252	267	63.72	71.97	67.07	68.73	65.02
P04196	254	269	74.97	80.71	80.82	83.57	80.75
P04196	256	267	77.88	85.82	89.81	83.28	79.04
P04196	256	269	66.12	70.64	68.53	68.48	65.64

P04196	256	270	63.26	66.39	69.25	75.53	67.79
P04196	256	271	80.36	86.71	95.85	95.99	87.7
P04196	265	289	34.7	36.32	42.96	52.62	68.57
P04196	270	303	48.81	63.53	64.41	70.4	70.68
P04196	282	315	51.12	58.61	65.84	72.06	76.35
P04196	284	303	88.38	94.69	95.69	97.69	93.9
P04196	284	312	40.81	47.54	54.79	57.44	64.22
P04196	296	324	33.99	37.41	44.45	56.16	73.26
P04196	301	335	57.26	69.98	79.44	85.76	90.68
P04196	306	320	79.91	88.08	90.6	90.16	92.13
P04196	307	337	27.95	31.88	40.01	46.89	54.33
P04196	312	328	11.85	15.13	20.99	24.27	30.7
P04196	312	334	91.88	96.23	98.47	96.29	94.53
P04196	326	352	52.45	58.41	71.64	72.47	76.16
P04196	340	365	41.79	46.83	57.76	72.63	74.71
P04196	343	364	44.64	52.04	63.53	74.61	82.61
P04196	350	377	39.23	44.15	54.64	71.8	76.07
P04196	352	369	22.85	30.35	40.83	45	55.52
P04196	353	370	22.52	31.88	44.1	49.15	59.95
P04196	367	396	50.65	61.01	65.57	71.41	77.92
P04196	411	440	51.8	57.28	67.36	78.11	78.16
P04196	459	476	28.12	31.27	43.61	59.56	76.68
P04196	459	477	28.49	34.13	47.3	61.72	77.07
P04196	469	486	34.01	35.88	44.5	47.88	51.88
P04196	471	482	21.94	23.21	25.8	51.79	71.79
P04196	478	499	98.66	101.53	103.68	102.52	100.09
P04196	478	500	95.39	95.41	100.57	100.57	96.81
P04196	478	501	101.47	104.63	106.2	106.3	103.1
P04196	489	517	32.97	37.04	45.87	56.91	73.09
P04196	489	518	33.01	36.35	46.21	57.18	73.41
P04196	491	525	48.95	53.6	65.09	75.89	77.98
P04196	493	522	40.63	46.15	53.28	56.31	62.53
P04196	498	522	37.91	40.58	44.25	49.48	55.35
P04196	502	514	14.93	20.36	29.27	31.14	43.12
P04196	509	517	87.91	100.87	104.66	103.85	101.15
P04196	509	518	72.19	82.3	87.33	94.83	96.21
P04196	510	517	86.87	98.51	96.16	101.46	97.16
P04196	511	517	83.81	97.87	101.87	99.33	97.74
P04196	513	518	90.38	100.94	102.79	101.35	93.12
P04196	518	525	80.98	94.64	103.55	102.59	98.04
P04196	519	525	85.99	99.98	108.73	107.65	103.56
P04196	520	525	81.88	97.21	105.2	102.58	98.47

Table S2. Observed structural adaptations of HRG in the presence of Zn^{2+}. Zinc binding induces conformational changes in HRG, likely promoting the compaction of the HRR, altering secondary and tertiary structure, and increasing solvent accessibility in certain regions. These changes may facilitate oligomer formation and enhance binding to interaction partners such as heparin sulfate and plasminogen. Note structural effects of zinc on high molecular weight kininogen (HK) have been included given the structural similarity of these proteins and their similar mechanisms of functional regulation.

Study	Protein(s) studied	Technique(s)	Observation/s upon Zn^{2+} addition	Interpretation
Herwald et al. (2001) *Eur. J. Biochem.* 268, 396-404.	Recombinant polypeptide corresponding to histidine-rich D5 region of HK.	Intrinsic fluorescence spectroscopy	Blue shift in fluorescence spectrum.	Compaction of the histidine-rich D5 region.
	Synthetic protein corresponding to full-length HK.	Negative-stain EM	HK appeared more spherical and compact. with reduced molecular	The binding of Zn^{2+} to domain D5 induces a conformational change in the entire HK molecule.

			diameter.	
Jancsó et al. (2009) *J. Inorg. Biochem.* 103, 1634-1643.	Synthetic oligopeptide fragment derived from the HRR of HRG.	Synchrotron radiation CD spectroscopy	Significant increase in intensity within the 195 to 230 nm range. Notably, in some cases, the emergence of positive band between 214 and 219 nm.	Conformational change in the peptide, possible polyproline-**II** to polyproline-**I** transition.
		^{1}H-NMR	Non-selective broadening of all NMR signals.	Formation of multiple Zn^{2+}-bound complexes, which are in intermediate mutual exchange with each other.
Vu et al. (2011) *J. Biol. Chem.* 286, 30314–30323.	Full-length HRG isolated from human plasma.	Intrinsic florescence spectroscopy	Decrease in fluorescence signal in presence of Zn^{2+}.	Changes in tertiary structure – potentially increased solvent exposure in regions surrounding tryptophan residues.
Martin et al. (2018) *Sci. Rep.* 8, 8646.	Synthetic peptide corresponding to HRR-PRR2.	CD spectroscopy	Altered far UV spectrum (decrease in minimum at 200 nm) – lack of defined	Changes in secondary structure of HRR – multiple configurational isomers.

			isodichrotic point.	
		^{1}H-NMR	Line broadening of His-sidechain (imidazole) signals upon Zn^{2+} titration.	Formation of multiple Zn^{2+}-complexes, which are in relatively slow (intermediate) mutual exchange with each other.
		nESI--TWIM-MS	Decrease in mean arrival time distribution, observed line broadening.	Molecular compaction with the formation of multiple Zn^{2+}-bound configurational isomers.
		nESI-MS/MS using collision-induced dissociation (CID) and electron transfer dissociation (ETD).	Reduced number of cleavages in CID and ETD spectra	Molecular compaction
Priebatsch et al. (2017) *FEBS Lett.* 591, 164-176.	intact HRG purified from human serum.	Limited proteolysis	Increased sensitivity to trypsin-mediated proteolytic cleavage in presence of Zn^{2+}.	Changes in secondary structure, increased solvent exposure and/or local unfolding in certain regions.
		Analytical size-exclusion	Increase in HRG monomer:dimer ratio.	Formation of dimer.

		chromatography		
Jones et al. (2004) *J. Biol. Chem.* 279, 30114–30122.	Recombinant proteins of full-length human HRG and the N1/N2 domain and synthetic oligopeptide fragments. corresponding to the HRR	Immunofluorescence flow cytometry and ELISA	N1/N2 domain bound specifically to immobilised heparin and cell-surface heparin sulfate. In contrast, synthetic peptide corresponding to the HRR did not bind cells. The binding of full length HRG but not the N1/N2 domain was potentiated in the presence of Zn^{2+}.	Zn^{2+} binding to the HRR facilitates structural change that enhances N1/N2 binding to heparins and/or that structural change facilitates oligomerisation increasing avidity of HRG for immobilised heparins.

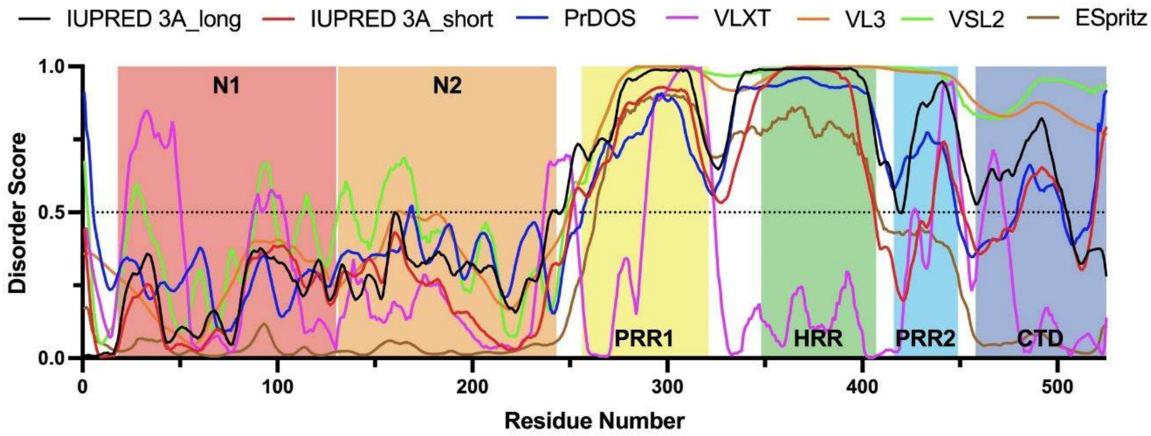

Figure S1. HRG predicted disorder using an eclectic range of computational predictors. Predicted disorder for IUPRED3A_long (black), IUPred3A_short (red), PrDOS (blue), VLXT (purple), VL3 (orange), VSL2 (green) and ESpritzX (brown). Regions with a disorder score above 0.5 are likely to be disordered in vivo.

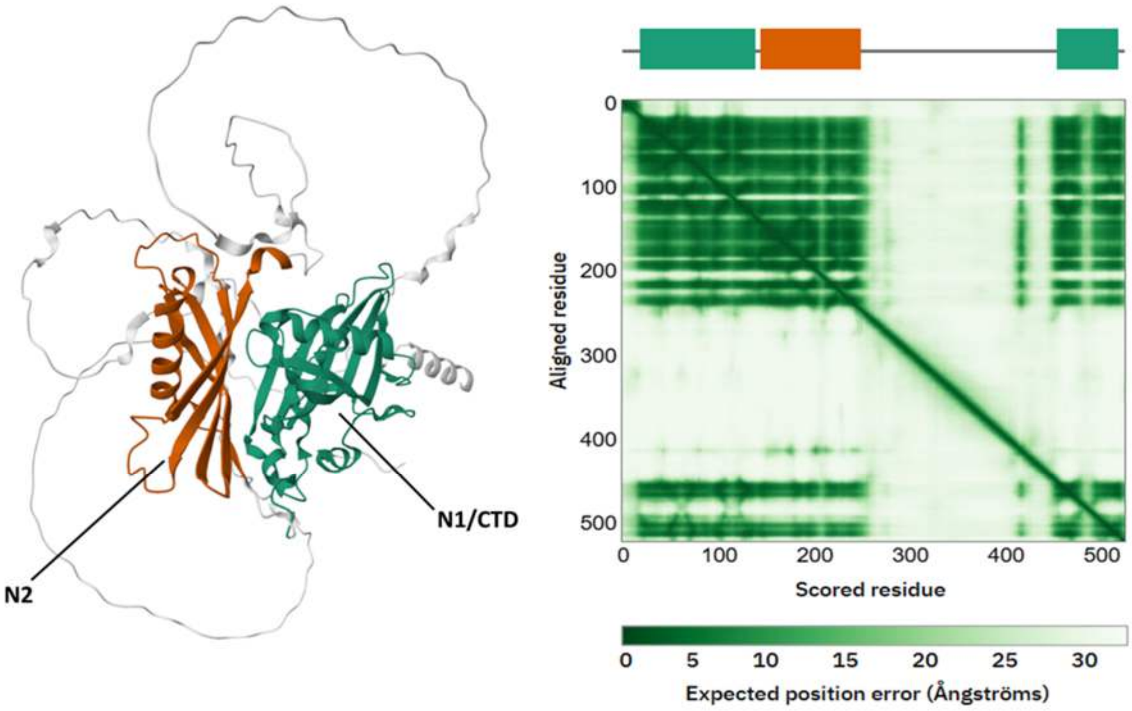

Figure S2. AlphaFold structural model for human HRG showing predicted alignment error (PAE) plot and The Encyclopaedia of Domains assignment. The N1/CTD and N2 domains are coloured green and orange respectively. Unannotated regions are shown in grey. AlphaFold assigns N1 and the CTD to the same structural superfamily (CATH 3.10.450.10, boundaries; 19-139, 455-519, Qscore; 73.68) suggesting that these regions, while discontinuous in sequence, act as a single functional/folding unit.

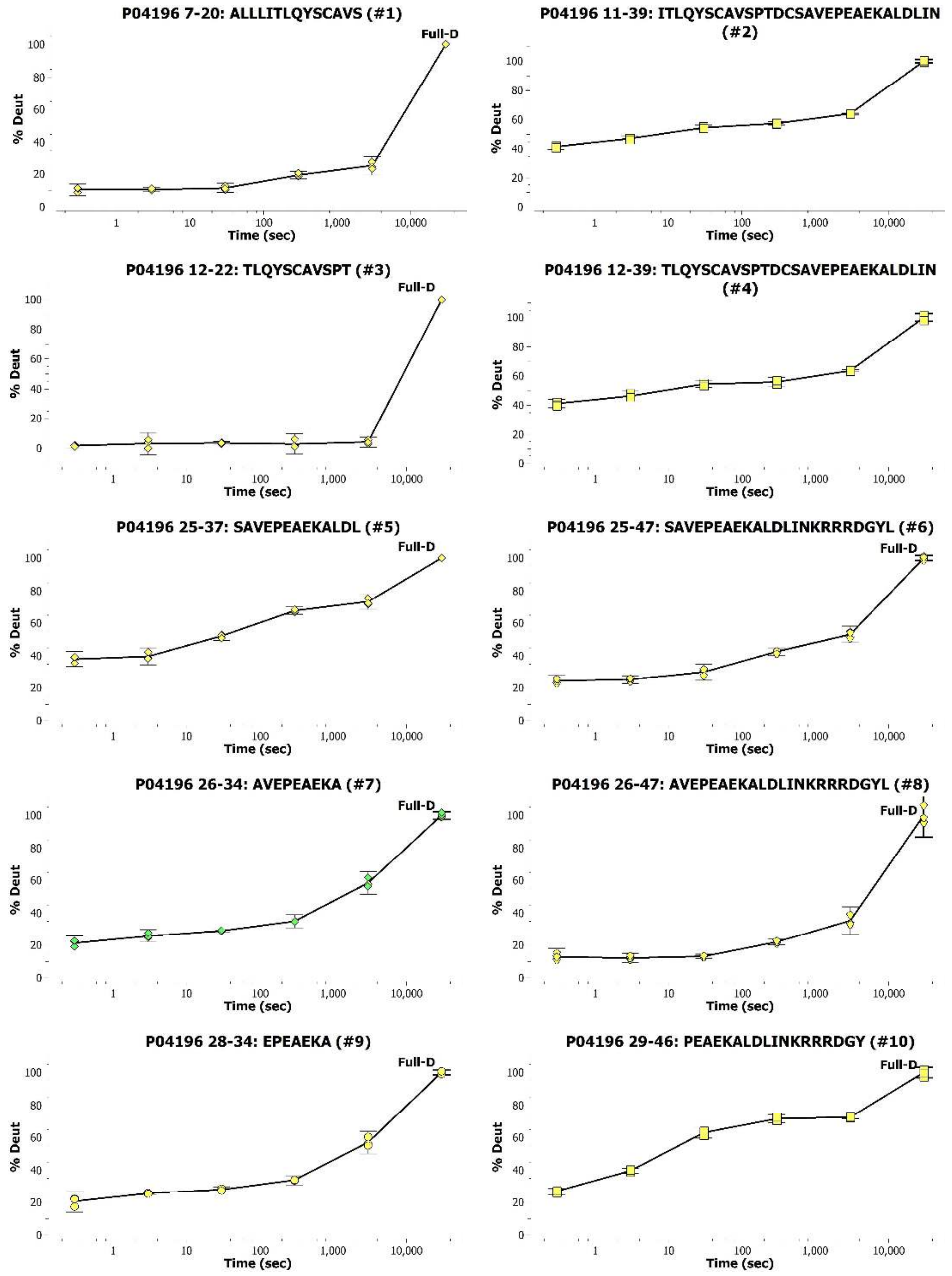
P04196 7-20: ALLLITLQYSCAVS (#1)
P04196 11-39: ITLQYSCAVSPTDCSAVEPEAEKALDLIN (#2)
P04196 12-22: TLQYSCAVSPT (#3)
P04196 12-39: TLQYSCAVSPTDCSAVEPEAEKALDLIN (#4)
P04196 25-37: SAVEPEAEKALDL (#5)
P04196 25-47: SAVEPEAEKALDLINKRRRDGYL (#6)
P04196 26-34: AVEPEAEKA (#7)
P04196 26-47: AVEPEAEKALDLINKRRRDGYL (#8)
P04196 28-34: EPEAEKA (#9)
P04196 29-46: PEAEKALDLINKRRRDGY (#10)
Full-D
% Deut
Time (sec)
0
20
40
60
80
100
1
10
100
1,000
10,000

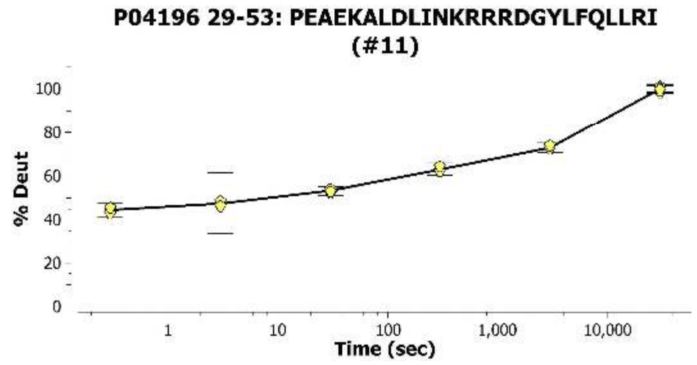
P04196 29-53: PEAEKALDLINKRRRDGYLFQLLRI (#11)
% Deut
Time (sec)

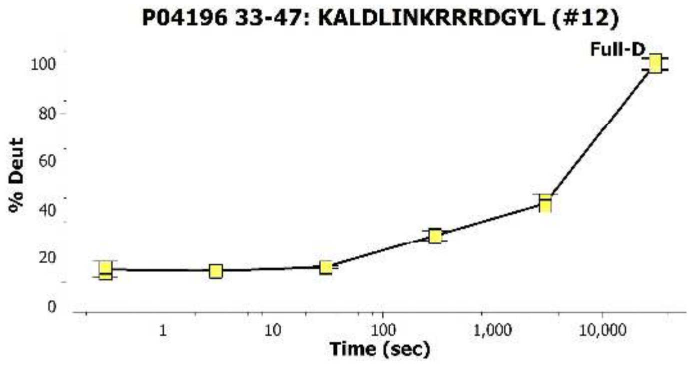
P04196 33-47: KALDLINKRRRDGYL (#12)
Full-D
% Deut
Time (sec)

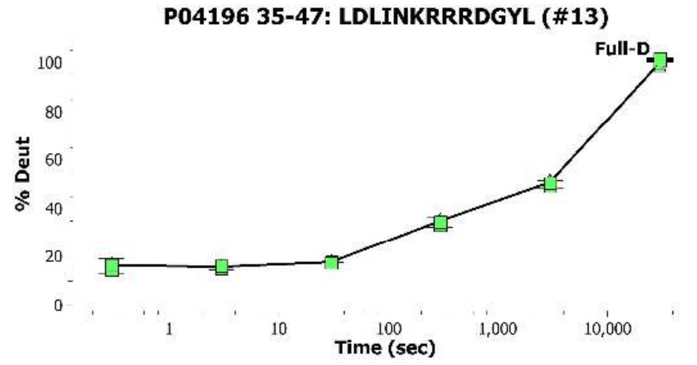
P04196 35-47: LDLINKRRRDGYL (#13)
Full-D
% Deut
Time (sec)

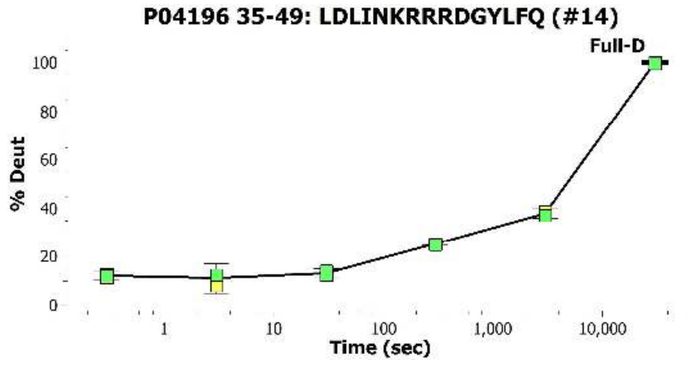
P04196 35-49: LDLINKRRRDGYLFQ (#14)
Full-D
% Deut
Time (sec)

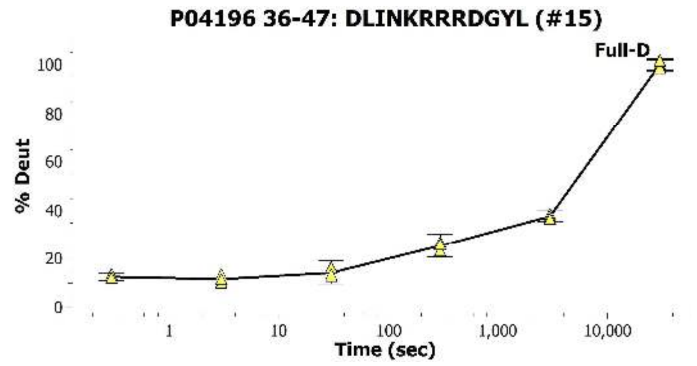
P04196 36-47: DLINKRRRDGYL (#15)
Full-D
% Deut
Time (sec)

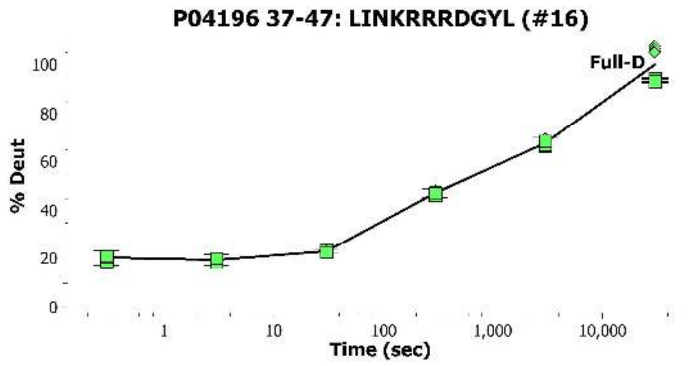
P04196 37-47: LINKRRRDGYL (#16)
Full-D
% Deut
Time (sec)

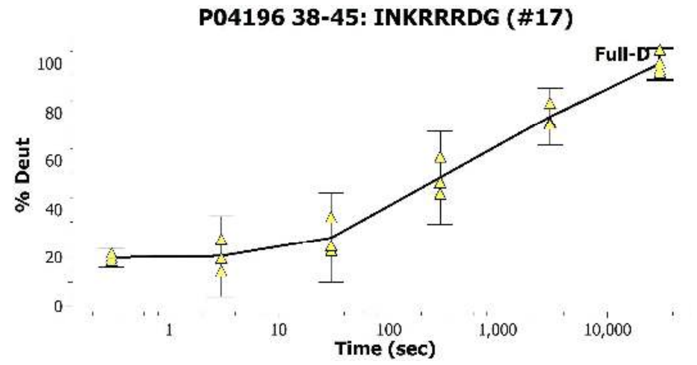
P04196 38-45: INKRRRDG (#17)
Full-D
% Deut
Time (sec)

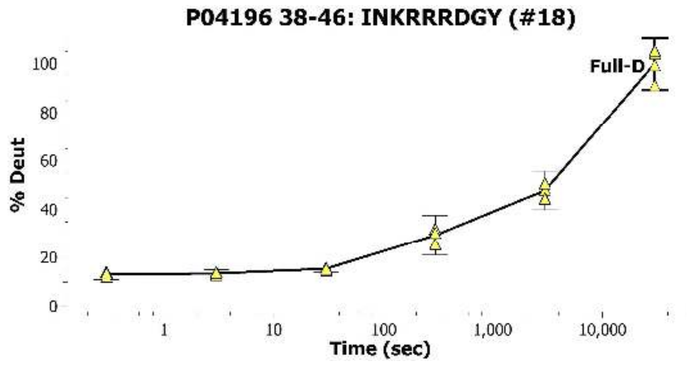
P04196 38-46: INKRRRDGY (#18)
Full-D
% Deut
Time (sec)

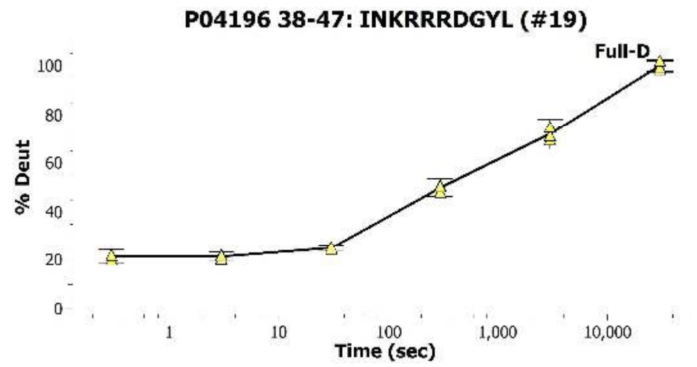
P04196 38-47: INKRRRDGYL (#19)
Full-D
% Deut
Time (sec)

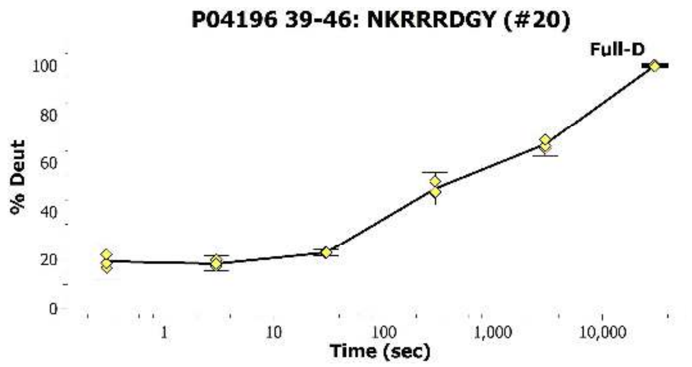
P04196 39-46: NKRRRDGY (#20)
Full-D
% Deut
Time (sec)

P04196 39-47: NKRRRDGYL (#21)

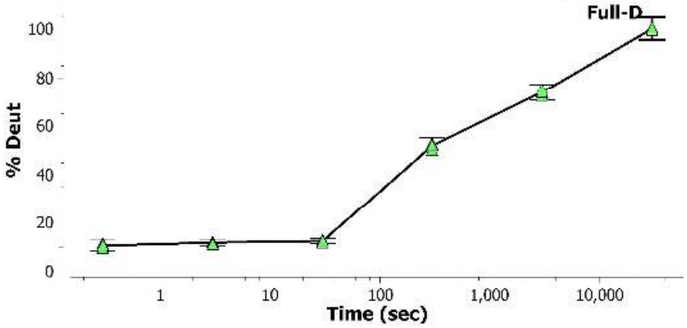

P04196 41-57: RRRDGYLFQLLRIADAH (#22)

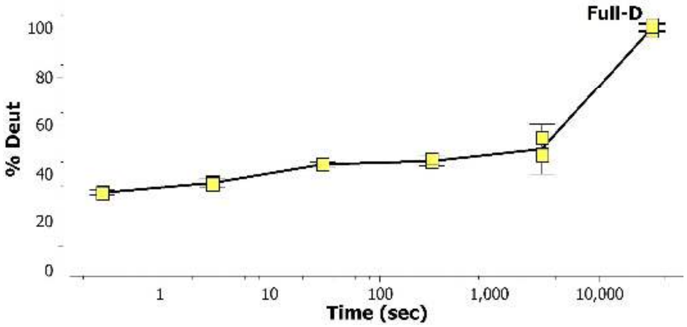

P04196 48-58: FQLLRIADAHL (#23)

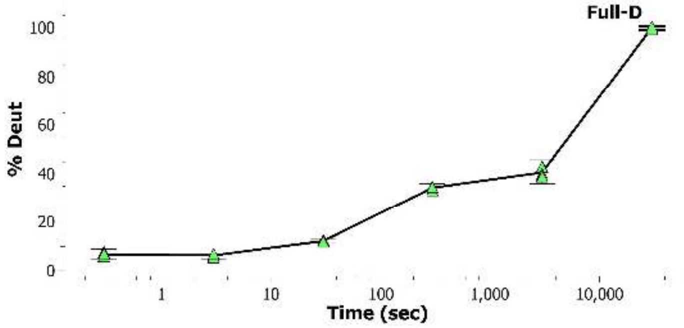

P04196 49-58: QLLRIADAHL (#24)

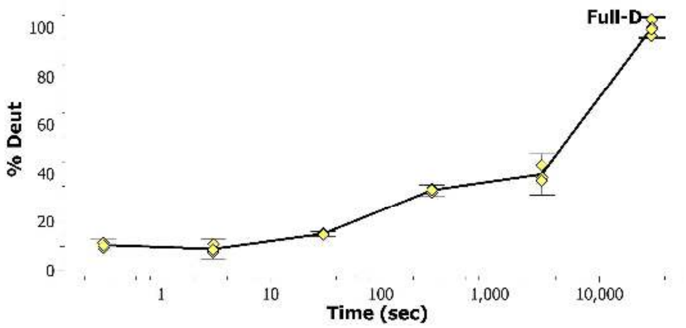

P04196 50-58: LLRIADAHL (#25)

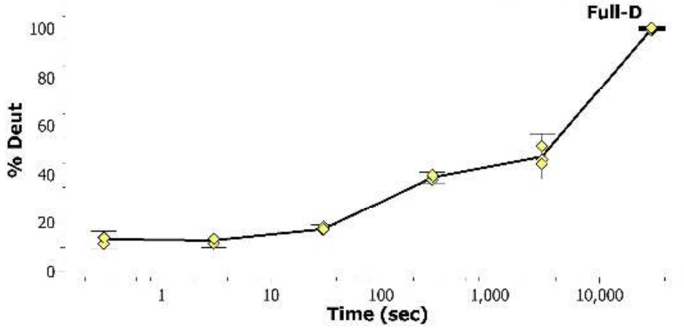

P04196 51-57: LRIADAH (#26)

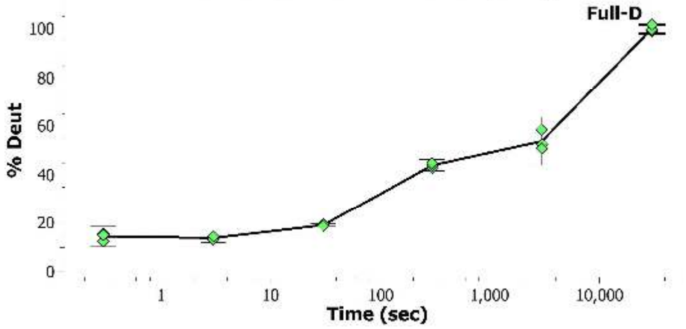

P04196 51-58: LRIADAHL (#27)

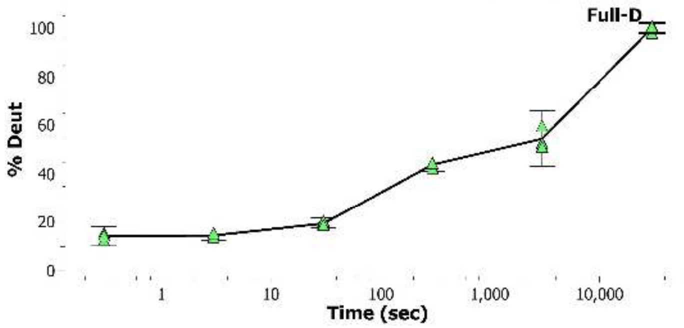

P04196 52-57: RIADAH (#28)

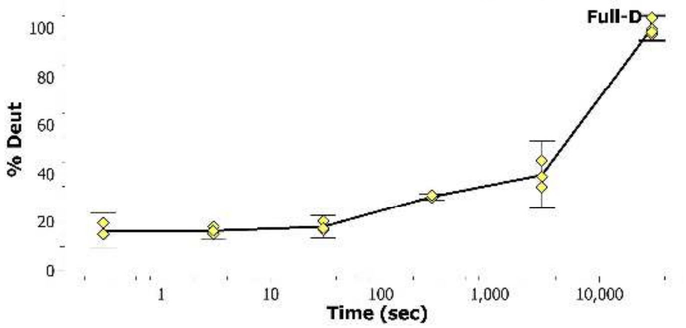

P04196 52-58: RIADAHL (#29)

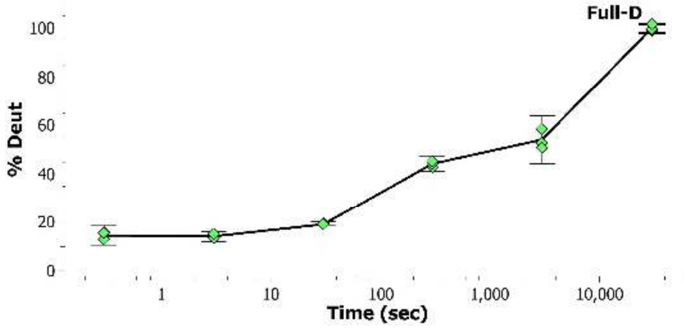

P04196 52-77: RIADAHLDRVENTTVYYLVLDVQESD (#30)

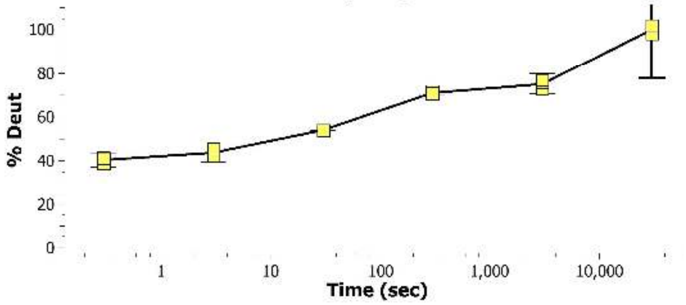

P04196 55-87: DAHLDRVENTTVYYLVLDVQESDCSVLSRKYWN (#31)

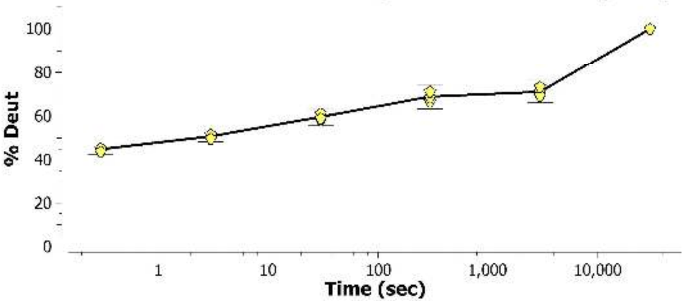

P04196 57-78: HLDRVENTTVYYLVLDVQESDC (#32)

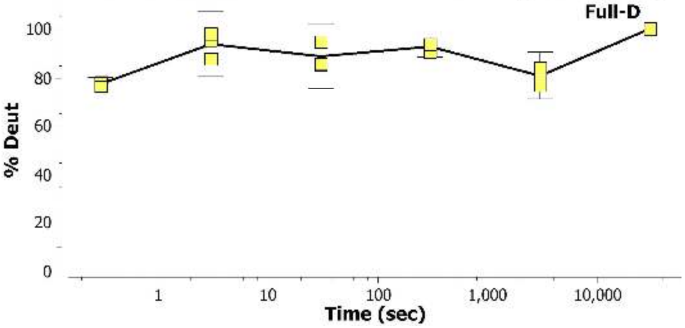

P04196 89-114: CEPPDSRRPSEIVIGQCKVIATRHSH (#33)

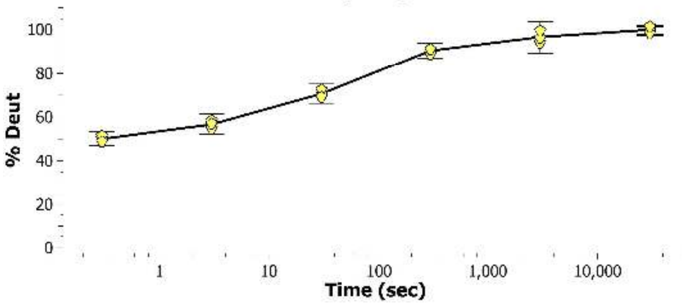

P04196 98-112: SEIVIGQCKVIATRH (#34)

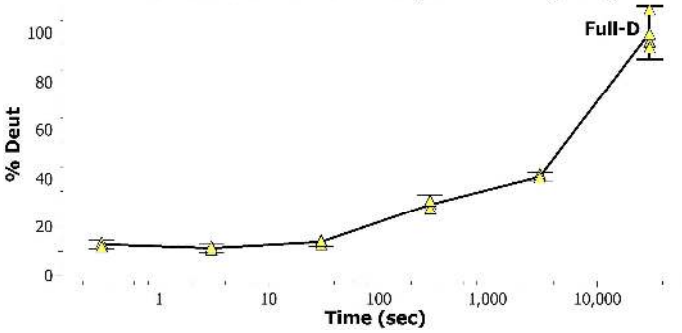

P04196 107-119: VIATRHSHESQDL (#35)

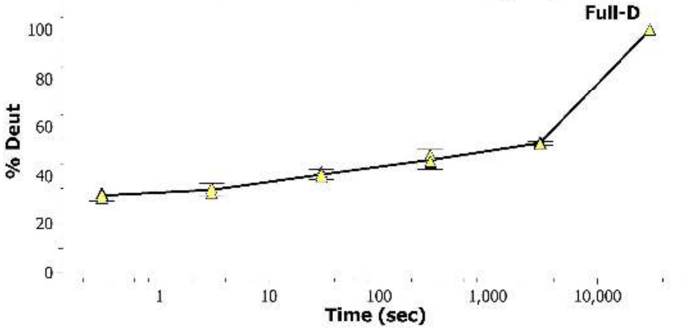

P04196 120-140: RVIDFNCTTSSVSSALANTKD (#36)

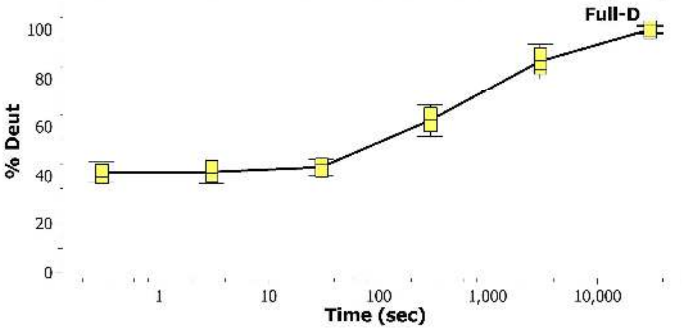

P04196 121-145: VIDFNCTTSSVSSALANTKDSPVLI (#37)

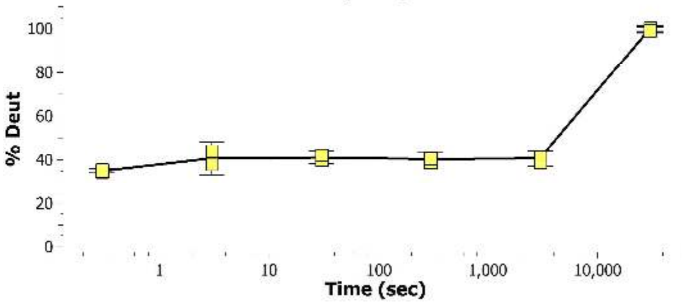

P04196 135-143: LANTKDSPV (#38)

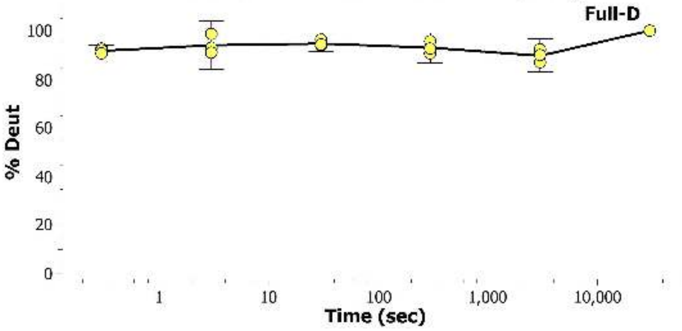

P04196 135-144: LANTKDSPVL (#39)

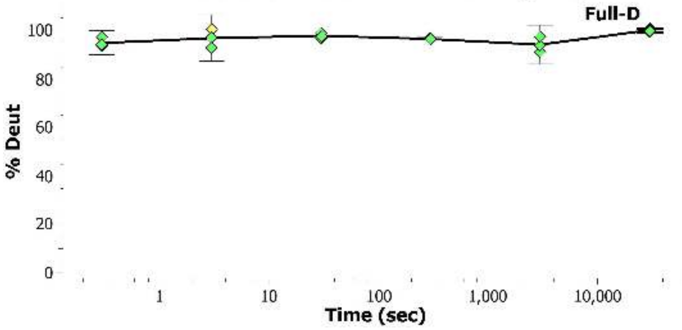

P04196 136-144: ANTKDSPVL (#40)

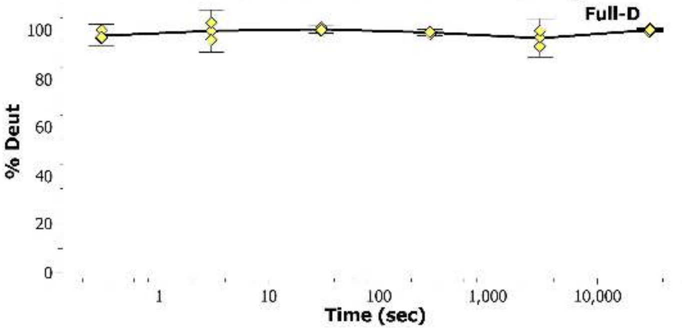

P04196 137-144: NTKDSPVL (#41)

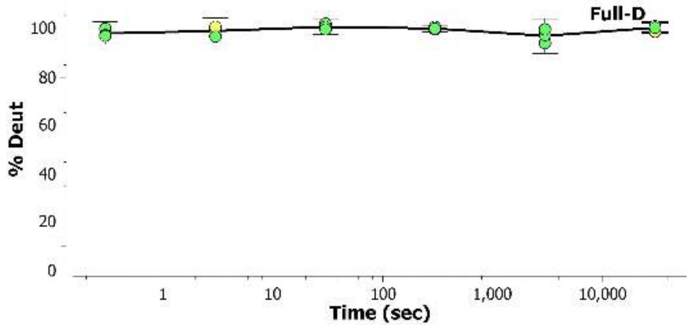

P04196 138-144: TKDSPVL (#42)

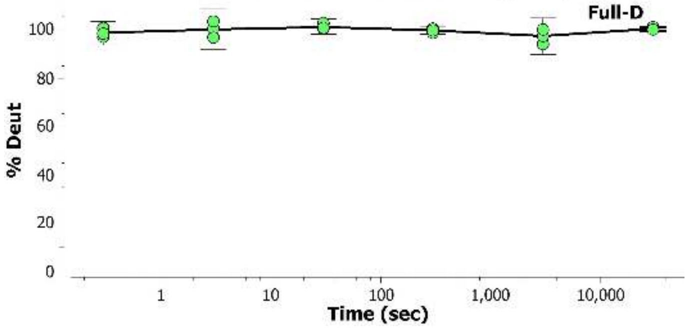

P04196 146-170: DFFEDTERYRKQANKALEKYKEEND (#43)

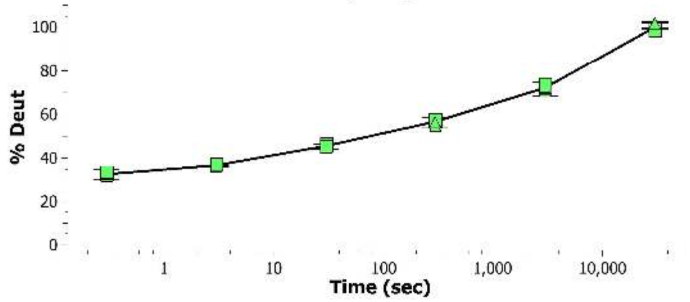

P04196 147-171: FFEDTERYRKQANKALEKYKEENDD (#44)

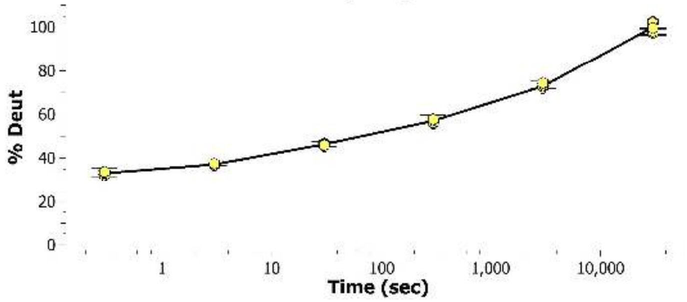

P04196 147-172: FFEDTERYRKQANKALEKYKEENDDF (#45)

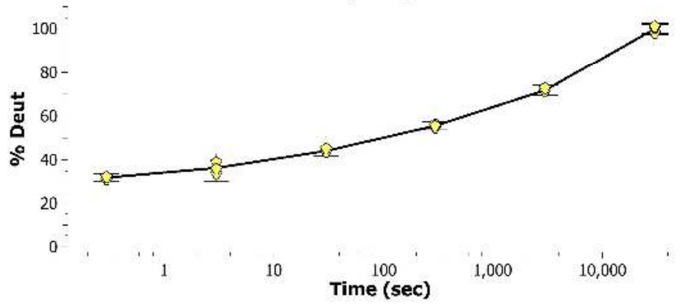

P04196 147-174: FFEDTERYRKQANKALEKYKEENDDFAS (#46)

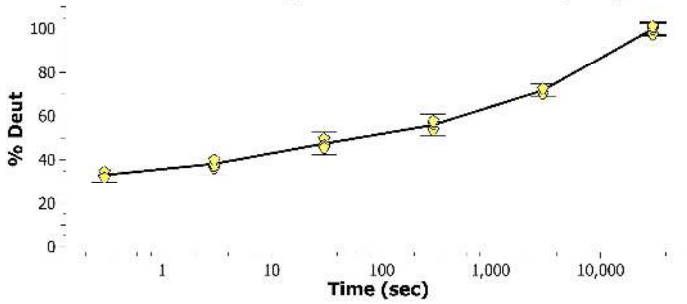

P04196 148-161: FEDTERYRKQANKA (#47)

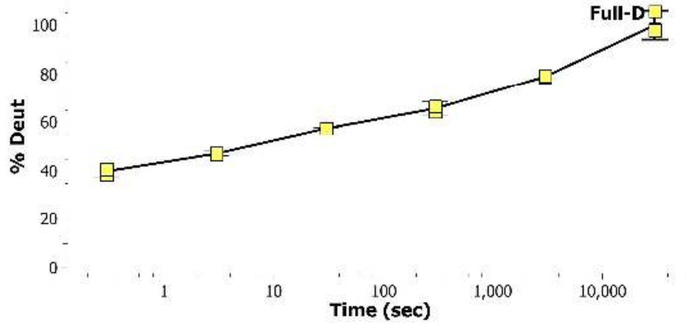

P04196 148-162: FEDTERYRKQANKAL (#48)

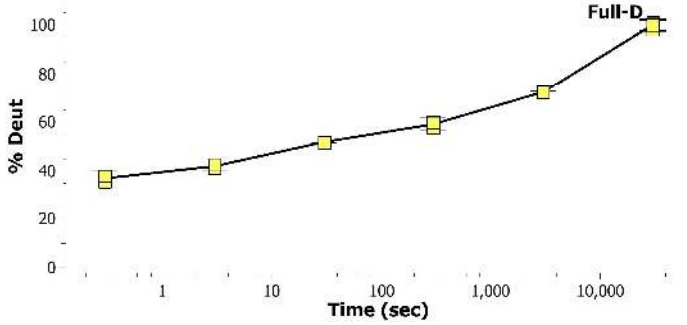

P04196 148-163: FEDTERYRKQANKALE (#49)

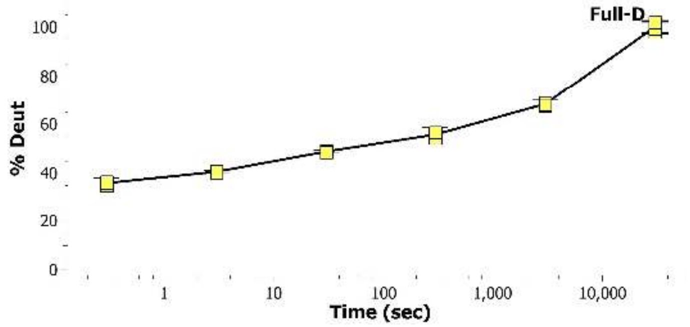

P04196 148-171: FEDTERYRKQANKALEKYKEENDD (#50)

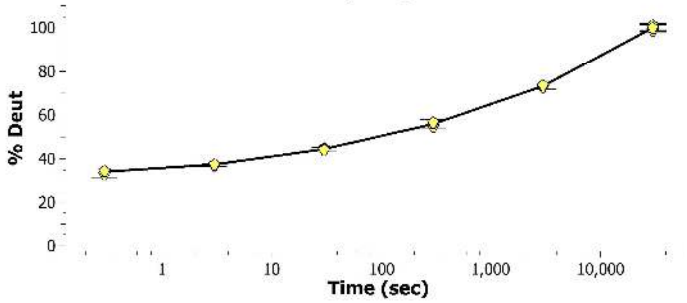

P04196 148-172: FEDTERYRKQANKALEKYKEENDDF (#51)

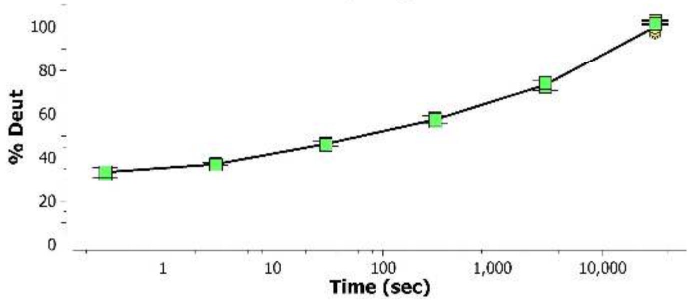

P04196 149-172: EDTERYRKQANKALEKYKEENDDF (#52)

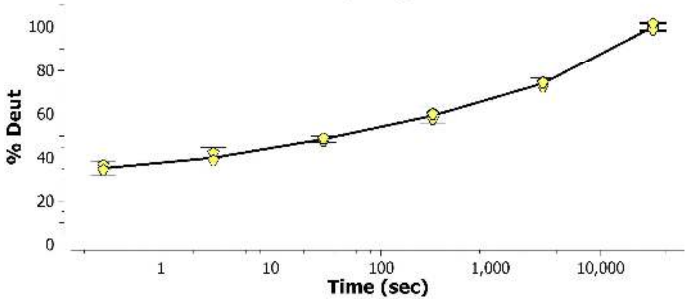

P04196 152-172: ERYRKQANKALEKYKEENDDF (#53)

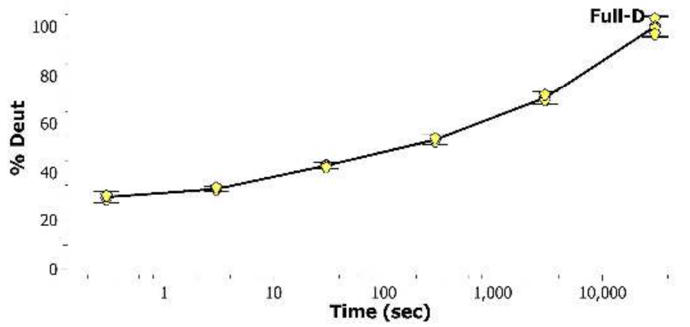

P04196 153-172: RYRKQANKALEKYKEENDDF (#54)

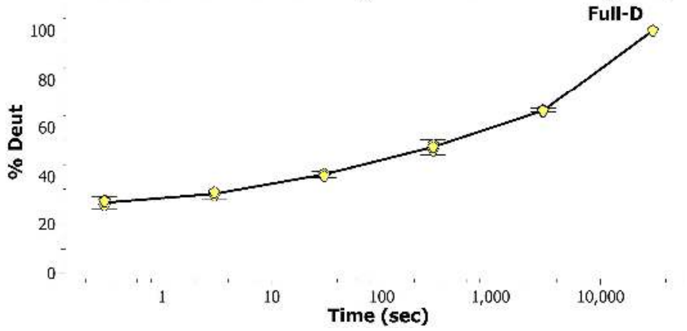

P04196 155-172: RKQANKALEKYKEENDDF (#55)

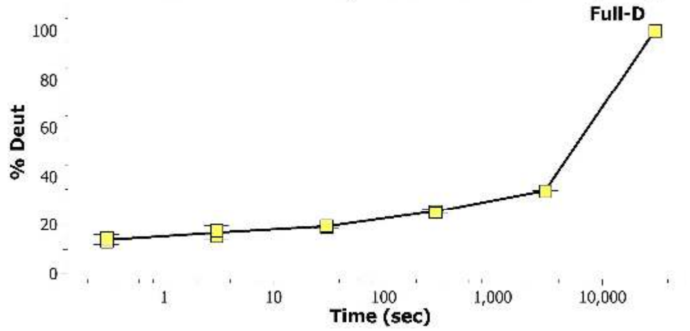

P04196 174-194: SFRVDRIERVARVRGGEGTGY (#56)

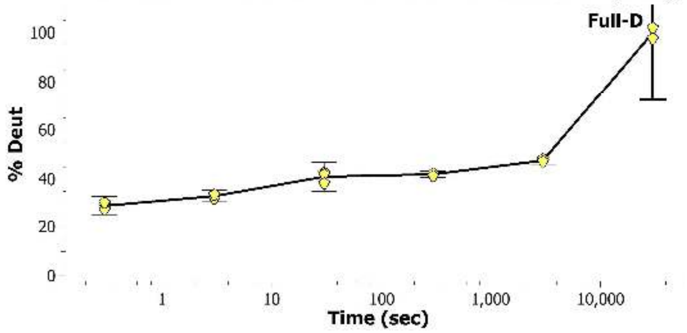

P04196 175-194: FRVDRIERVARVRGGEGTGY (#57)

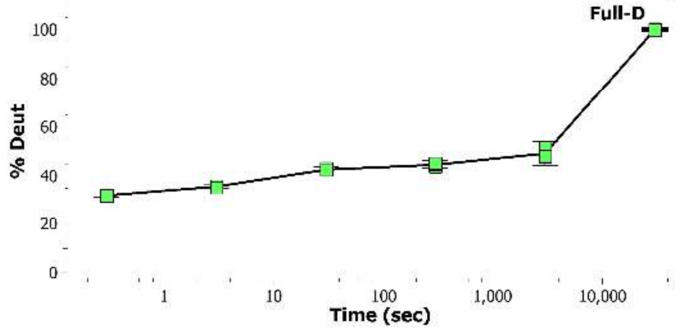

P04196 175-195: FRVDRIERVARVRGGEGTGYF (#58)

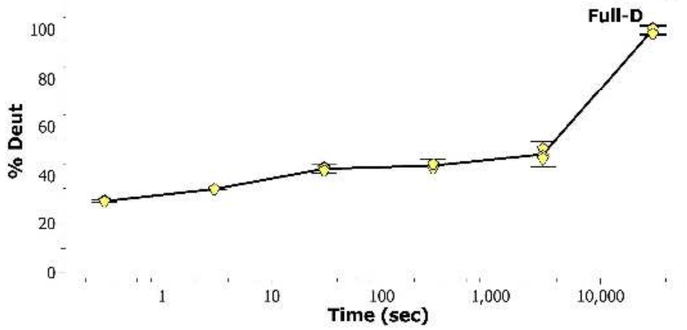

P04196 176-183: RVDRIERV (#59)

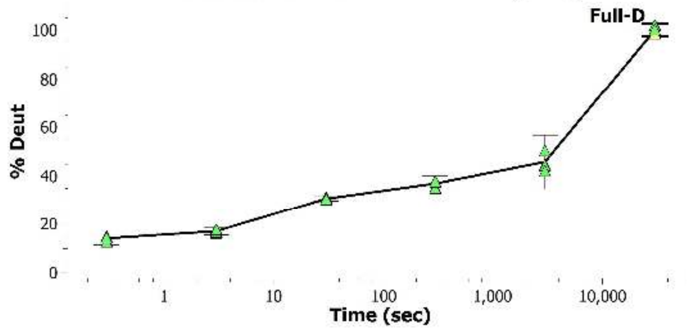

P04196 176-194: RVDRIERVARVRGGEGTGY (#60)

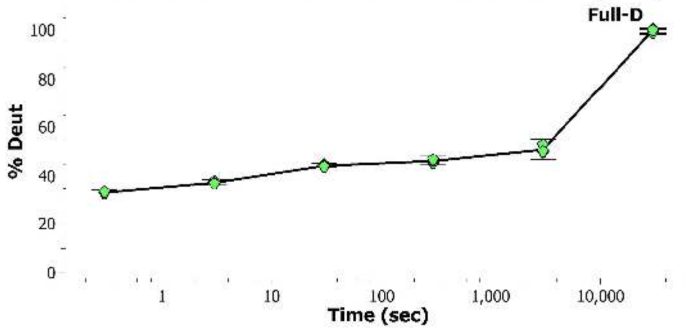

P04196 176-195: RVDRIERVARVRGGEGTGYF (#61)

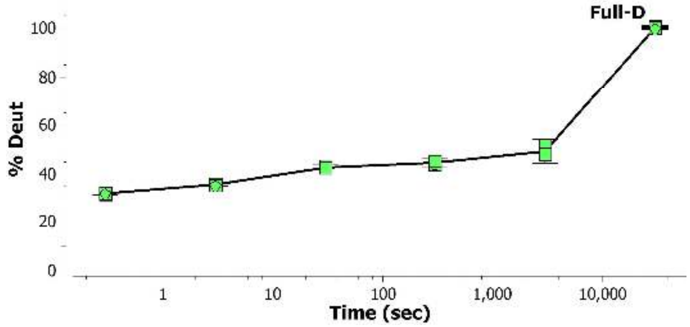

P04196 176-196: RVDRIERVARVRGGEGTGYFV (#62)

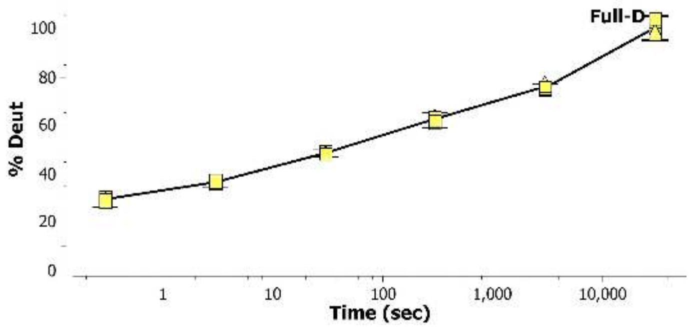

P04196 185-209: RVRGGEGTGYFVDFSVRNCPRHHFP (#63)

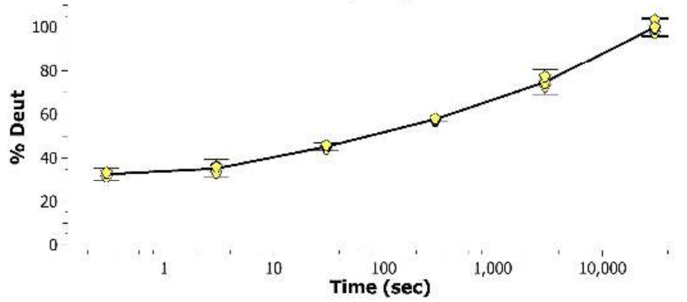

P04196 187-202: RGGEGTGYFVDFSVRN (#64)

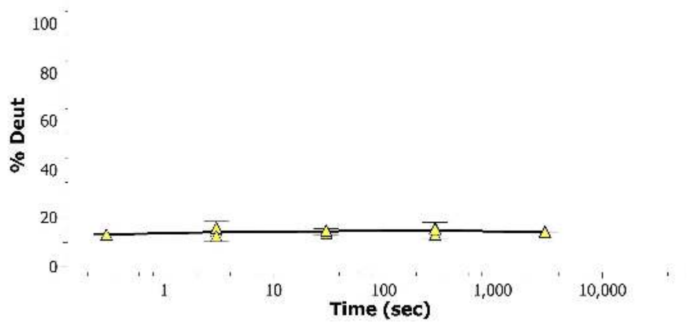

P04196 194-205: YFVDFSVRNCPR (#65)

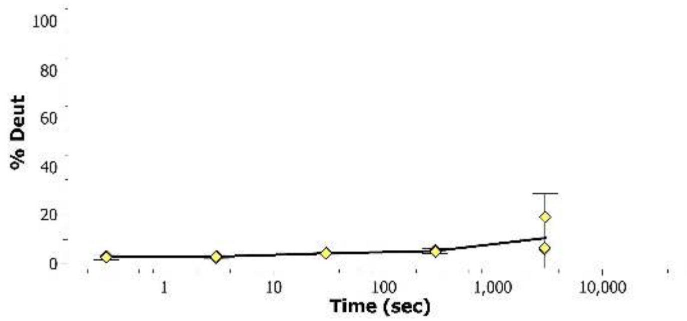

P04196 202-228: NCPRHHFPRHPNVFGFCRADLFYDVEA (#66)

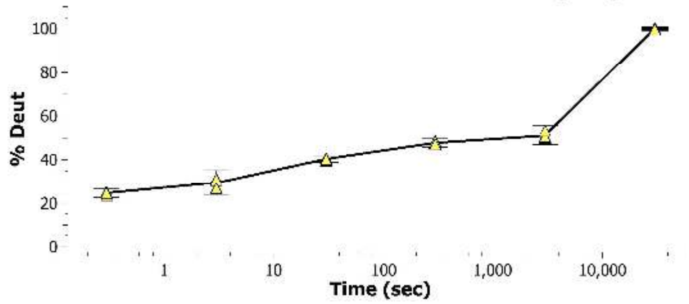

P04196 210-227: RHPNVFGFCRADLFYDVE (#67)

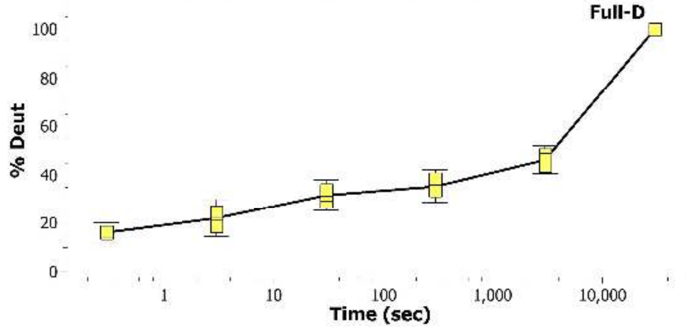

P04196 222-235: LFYDVEALDLESPK (#68)

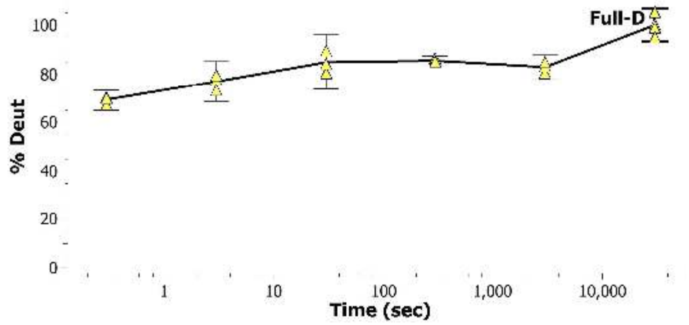

P04196 223-228: FYDVEA (#69)

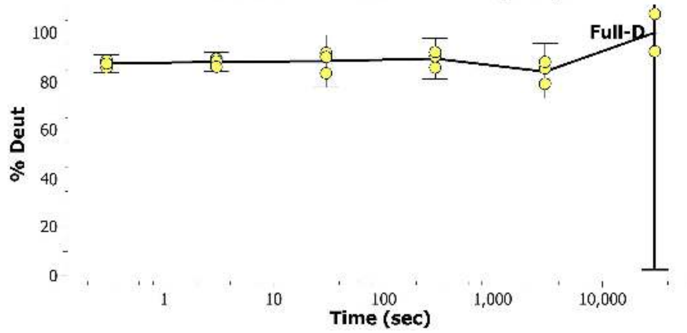

P04196 227-254: EALDLESPKNLVINCEVFDPQEHENING (#70)

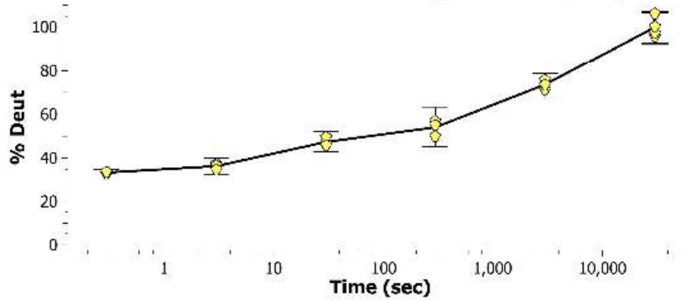

P04196 228-236: ALDLESPKN (#71)

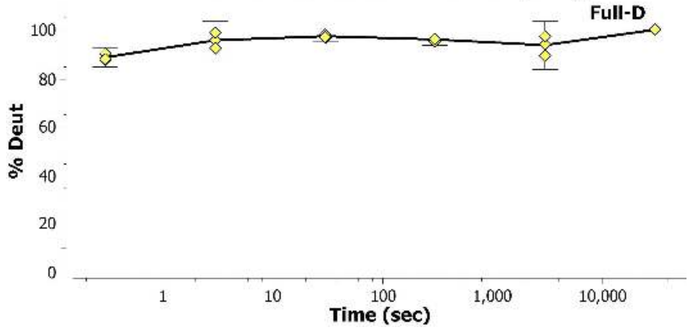

P04196 229-236: LDLESPKN (#72)

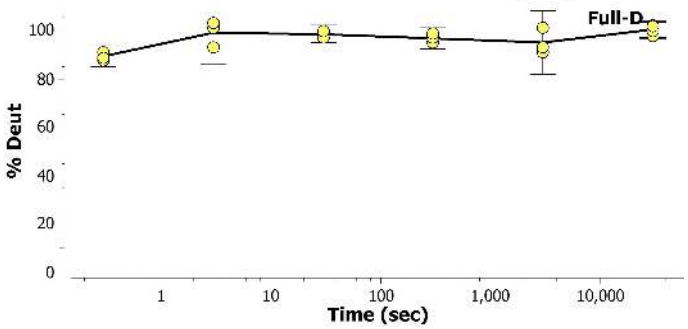

P04196 230-236: DLESPKN (#73)

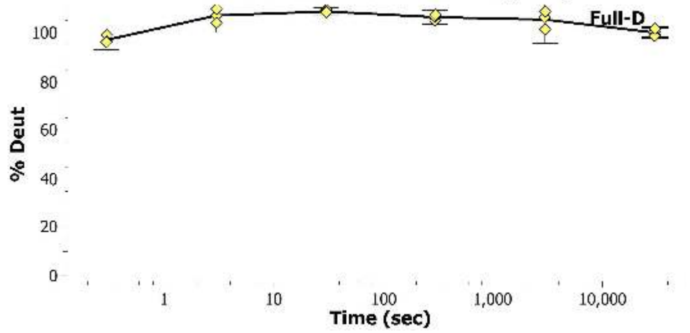

P04196 231-236: LESPKN (#74)

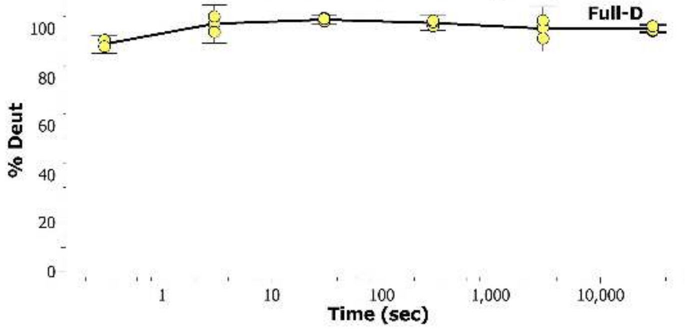

P04196 236-253: NLVINCEVFDPQEHENIN (#75)

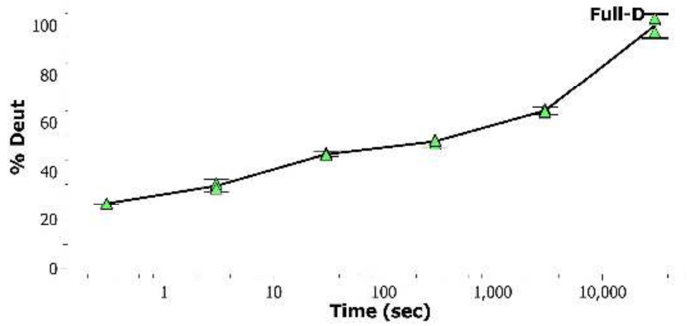

P04196 237-262: LVINCEVFDPQEHENINGVPPHLGHP (#76)

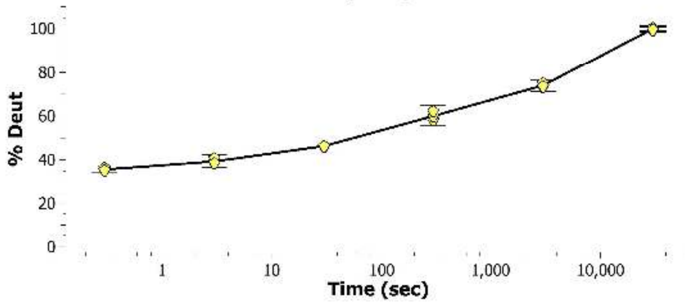

P04196 241-266: CEVFDPQEHENINGVPPHLGHPFHWG (#77)

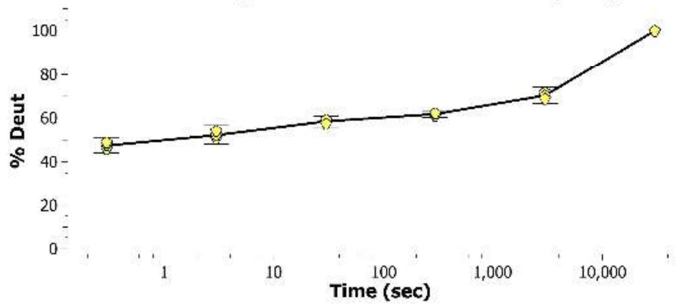

P04196 243-250: VFDPQEHE (#78)

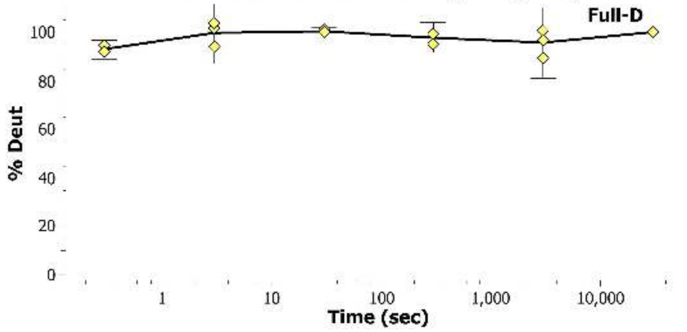

P04196 243-267: VFDPQEHENINGVPPHLGHPFHWGG (#79)

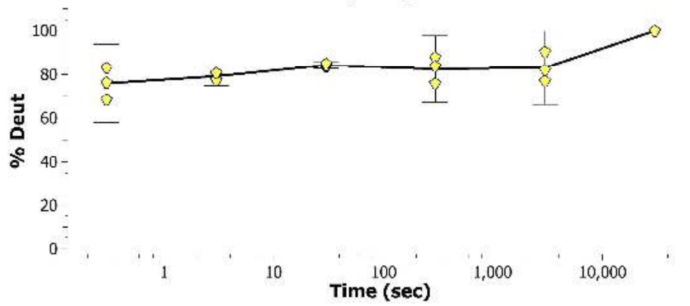

P04196 248-268: EHENINGVPPHLGHPFHWGGH (#80)

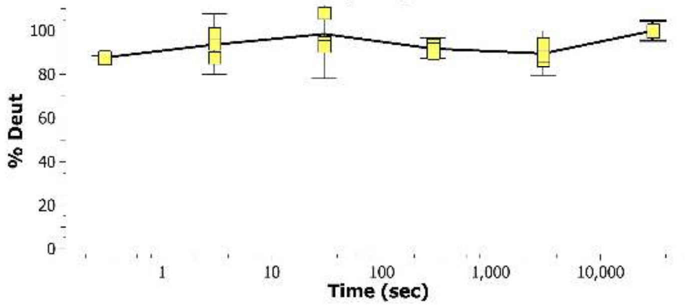

P04196 248-271: EHENINGVPPHLGHPFHWGGHERS (#81)

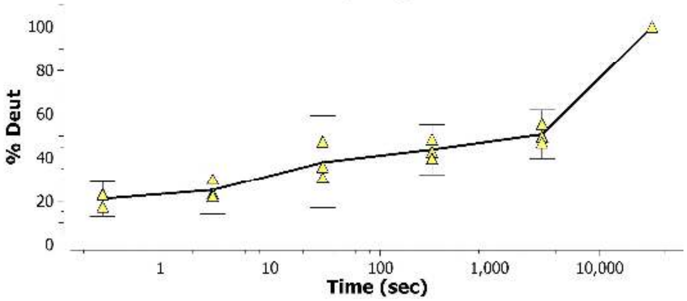

P04196 249-261: HENINGVPPHLGH (#82)

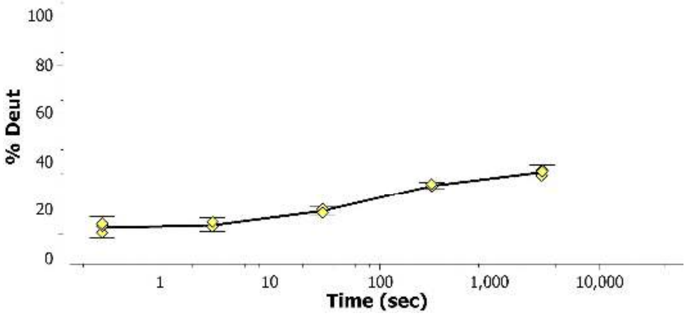

P04196 251-267: NINGVPPHLGHPFHWGG (#83)

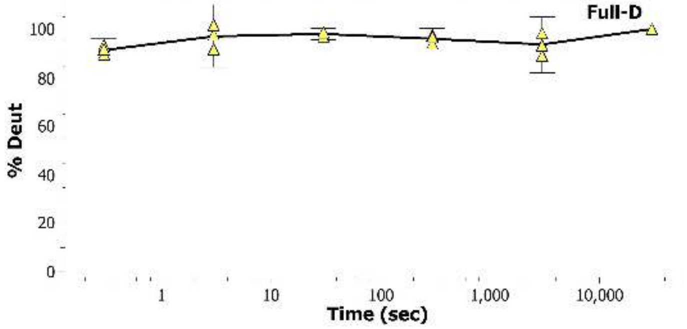

P04196 251-269: NINGVPPHLGHPFHWGGHE (#84)

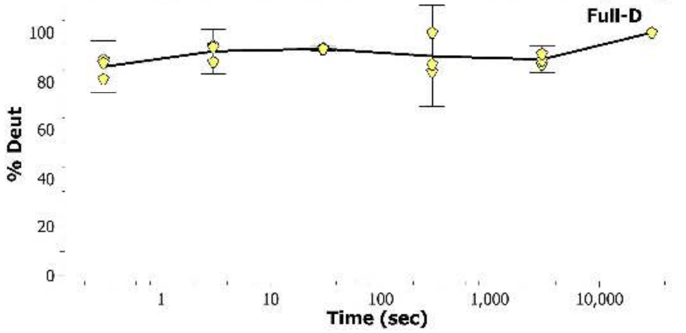

P04196 251-271: NINGVPPHLGHPFHWGGHERS (#85)

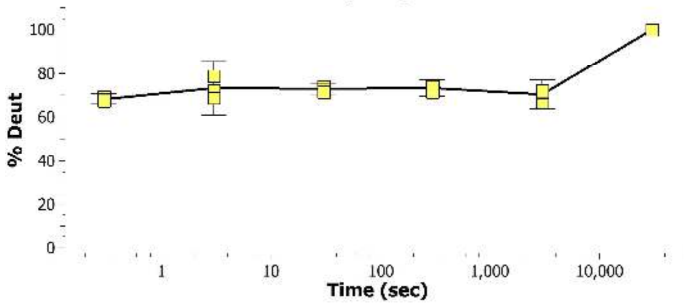

P04196 252-267: INGVPPHLGHPFHWGG (#86)

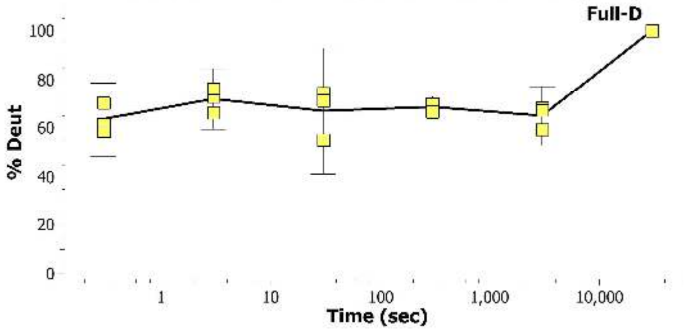

P04196 254-269: GVPPHLGHPFHWGGHE (#87)

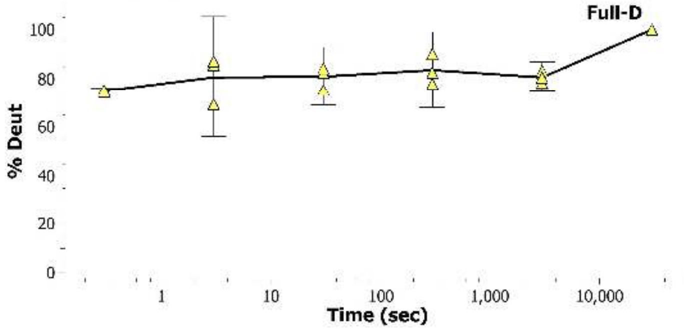

P04196 256-267: PPHLGHPFHWGG (#88)

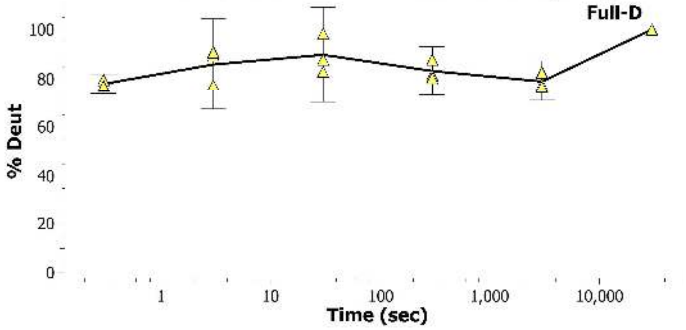

P04196 256-269: PPHLGHPFHWGGHE (#89)

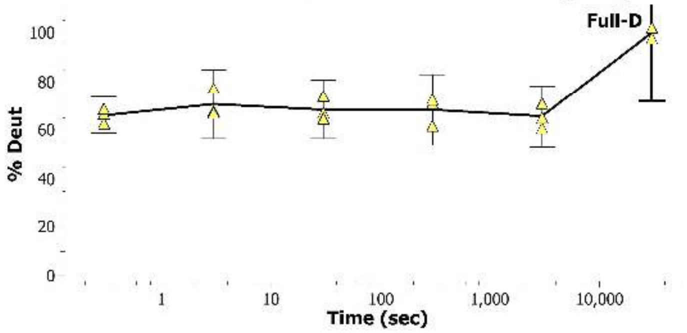

P04196 256-270: PPHLGHPFHWGGHER (#90)

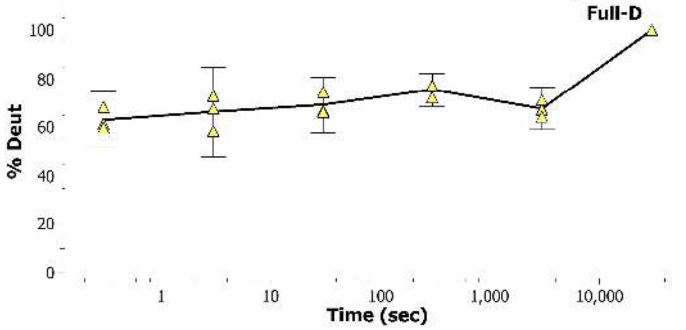

P04196 256-271: PPHLGHPFHWGGHERS (#91)

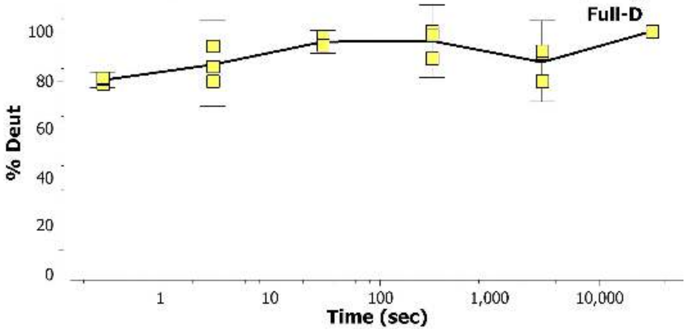

P04196 265-289: WGGHERSSTTKPPFKPHGSRDHHHP (#92)

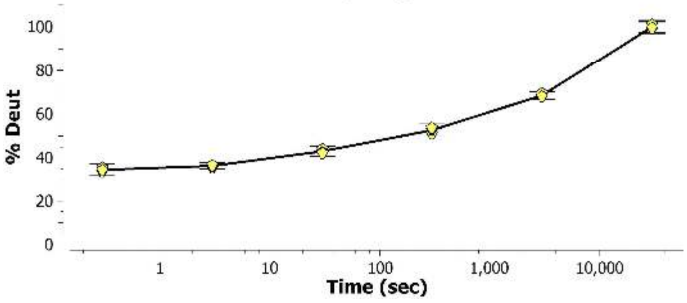

P04196 270-303: RSSTTKPPFKPHGSRDHHHPHKPHEHGPPPPPDE (#93)

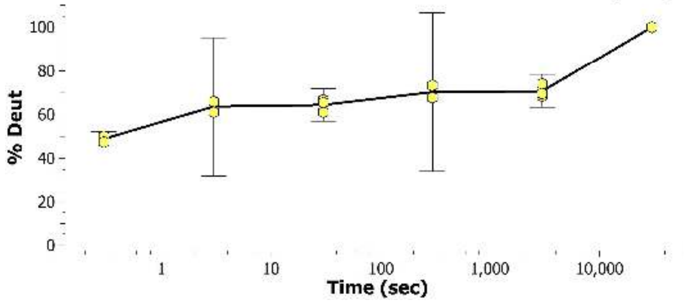

P04196 282-315: GSRDHHHPHKPHEHGPPPPPDERDHSHGPPLPQG (#94)

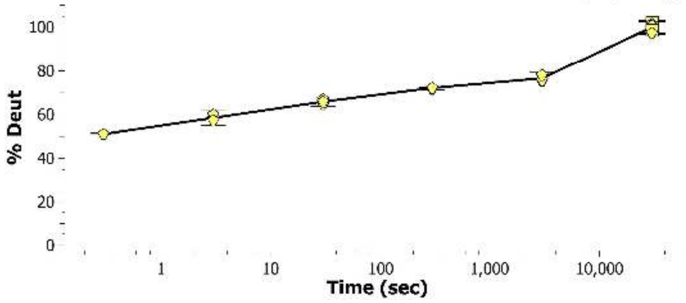

P04196 284-303: RDHHHPHKPHEHGPPPPPDE (#95)

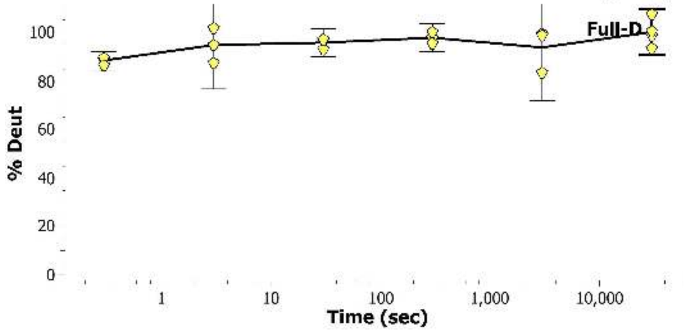

P04196 284-312: RDHHHPHKPHEHGPPPPPDERDHSHGPPL (#96)

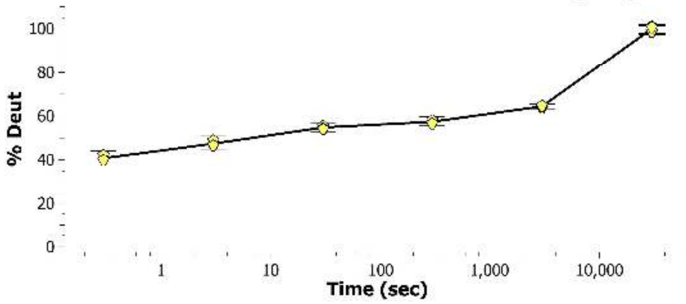

P04196 296-324: GPPPPPDERDHSHGPPLPQGPPPLLPMSC (#97)

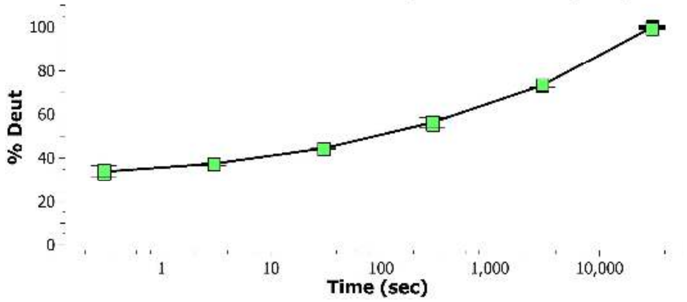

P04196 301-335: PDERDHSHGPPLPQGPPPLLPMSCSSCQHATFGTN (#98)

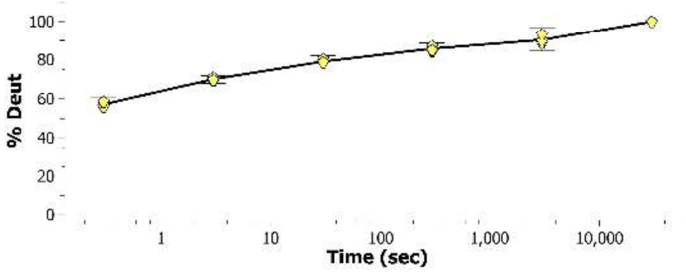

P04196 306-320: HSHGPPLPQGPPPLL (#99)

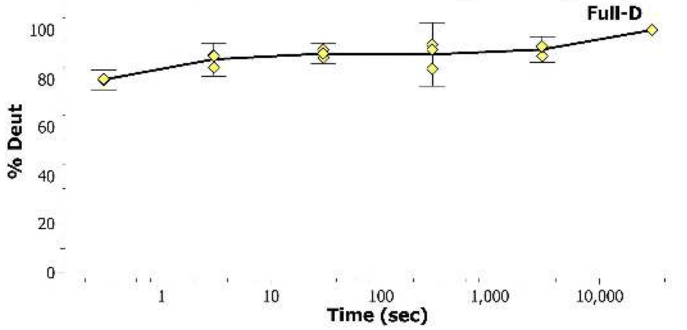

P04196 307-337: SHGPPLPQGPPPLLPMSCSSCQHATFGTNGA (#100)

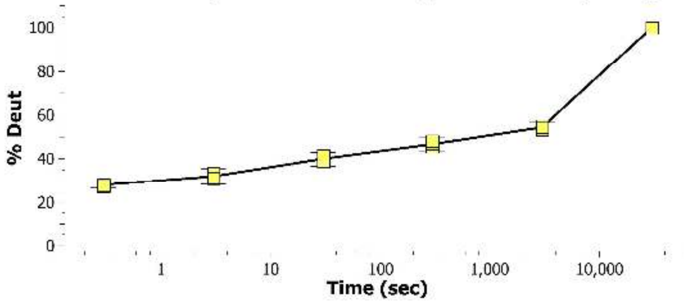

P04196 312-328: LPQGPPPLLPMSCSSCQ (#101)

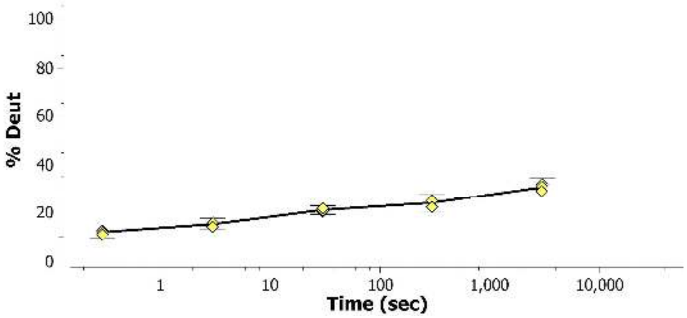

P04196 312-334: LPQGPPPLLPMSCSSCQHATFGT (#102)

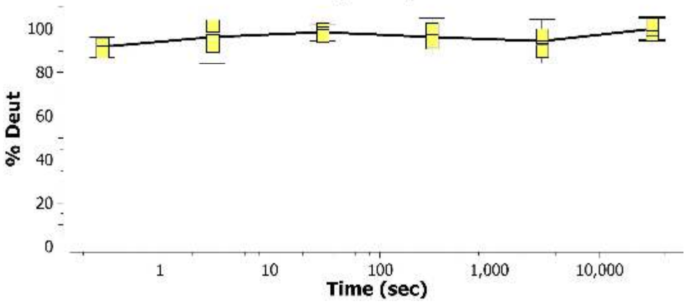

P04196 326-352: SCQHATFGTNGAQRHSHNNNSSDLHPH (#103)

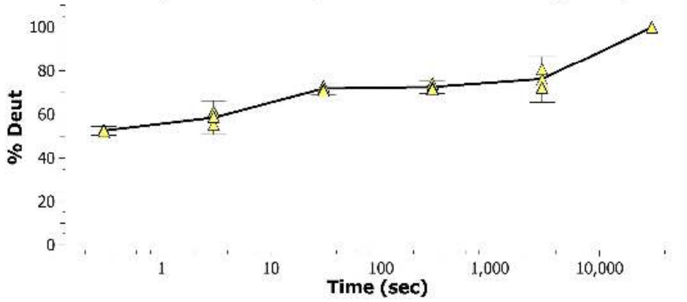

P04196 340-365: HSHNNNSSDLHPHKHHSHEQHPHGHH (#104)

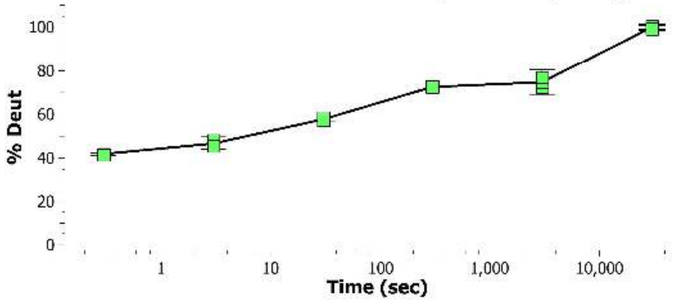

P04196 343-364: NNNSSDLHPHKHHSHEQHPHGH (#105)

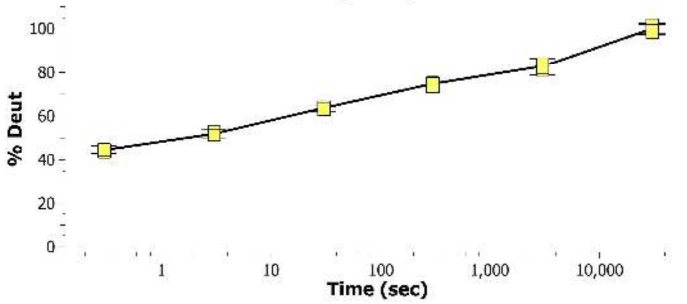

P04196 350-377: HPHKHHSHEQHPHGHHPHAHHPHEHDTH (#106)

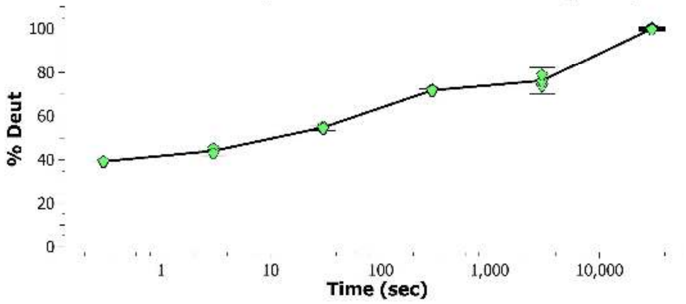

P04196 352-369: HKHHSHEQHPHGHHPHAH (#107)

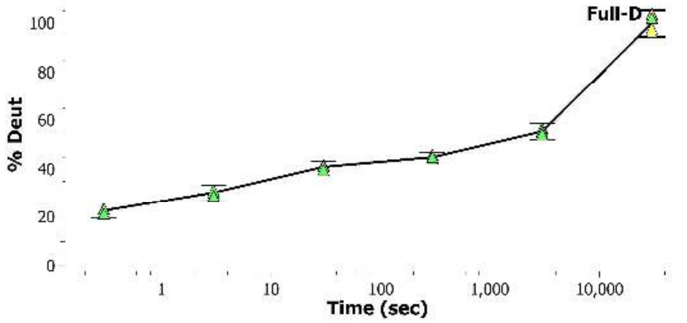

P04196 353-370: KHHSHEQHPHGHHPHAHH (#108)

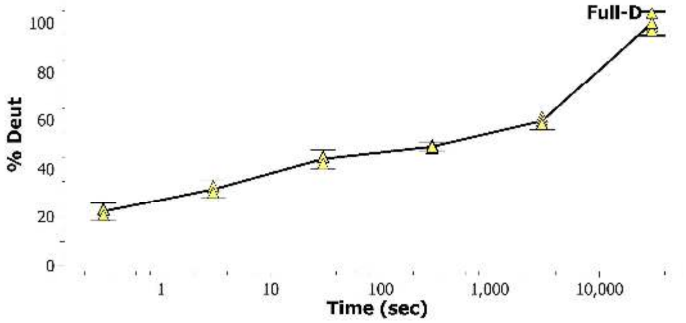

P04196 367-396: HAHHPHEHDTHRQHPHGHHPHGHHPHGHHP (#109)

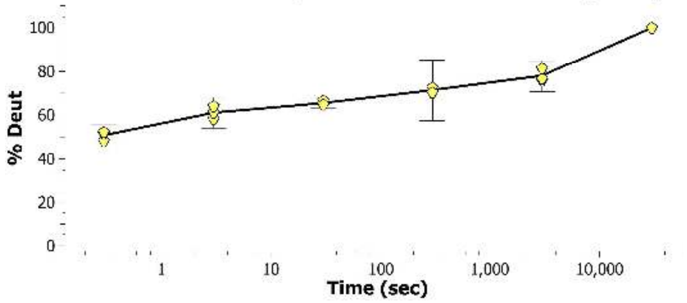

P04196 411-440: FQDYGPCDPPPHNQGHCCHGHGPPPGHLRR (#110)

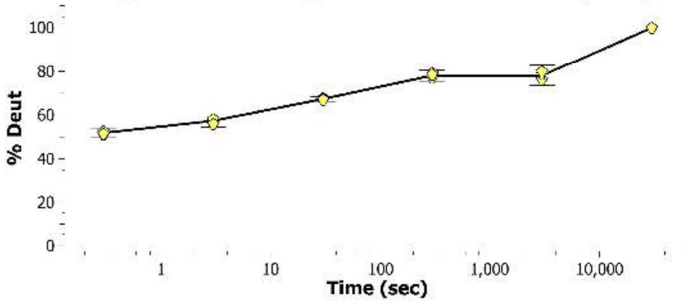

P04196 459-476: YRLPPLRKGEVLPLPEAN (#111)

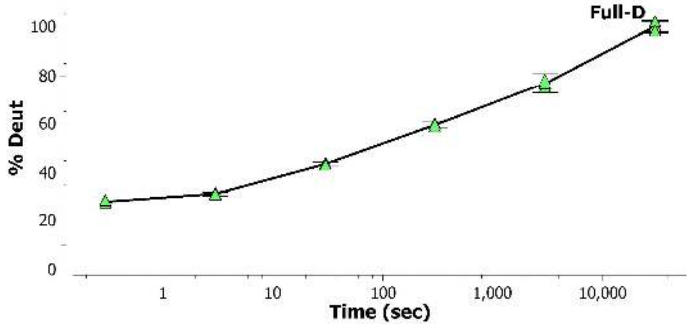

P04196 459-477: YRLPPLRKGEVLPLPEANF (#112)

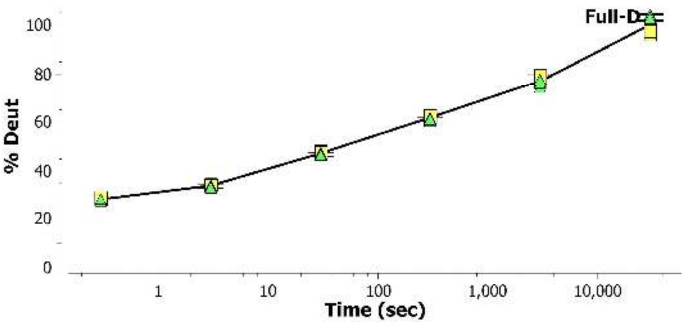

P04196 469-486: VLPLPEANFPSFPLPHHK (#113)

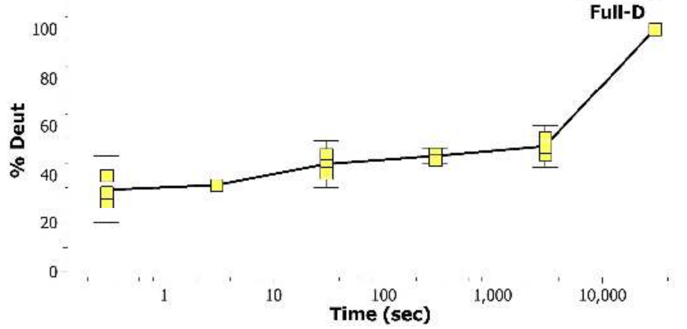

P04196 471-482: PLPEANFPSFPL (#114)

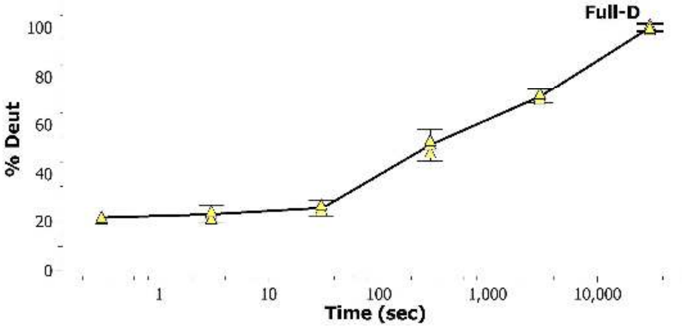

P04196 478-499: PSFPLPHHKHPLKPDNQPFPQS (#115)

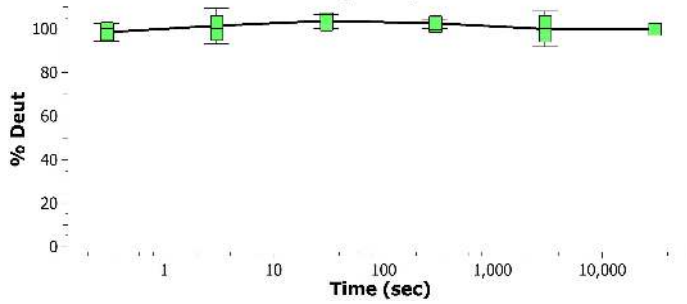

P04196 478-500: PSFPLPHHKHPLKPDNQPFPQSV (#116)

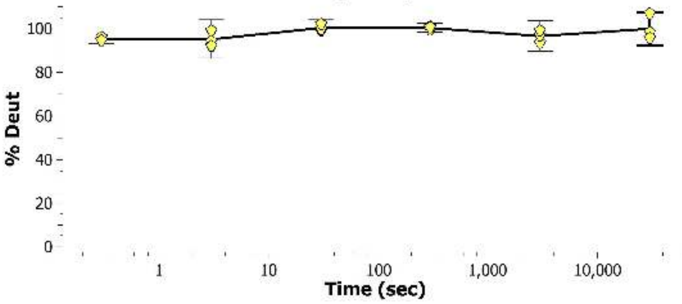

P04196 478-501: PSFPLPHHKHPLKPDNQPFPQSVS (#117)

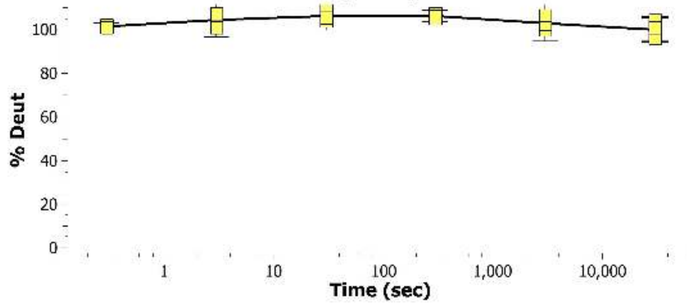

P04196 489-517: LKPDNQPFPQSVSESCPGKFKSGFPQVSM (#118)

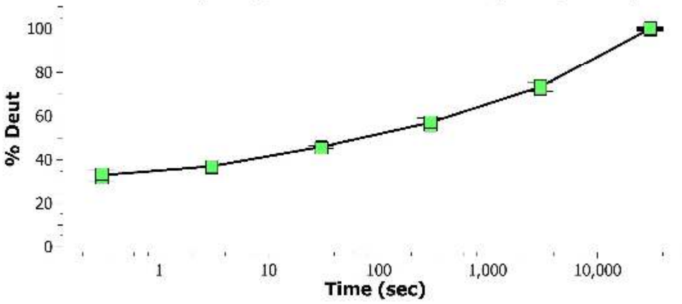

P04196 489-518: LKPDNQPFPQSVSESCPGKFKSGFPQVSMF (#119)

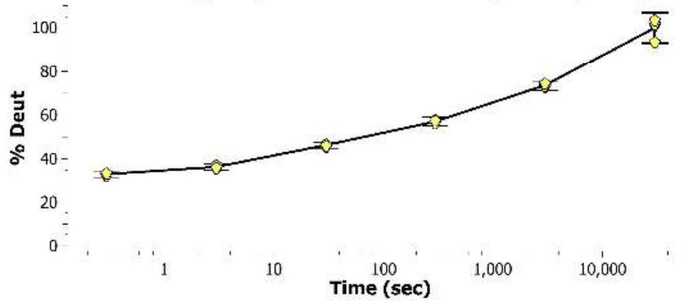

P04196 491-525: PDNQPFPQSVSESCPGKFKSGFPQVSMFFTHTFPK (#120)

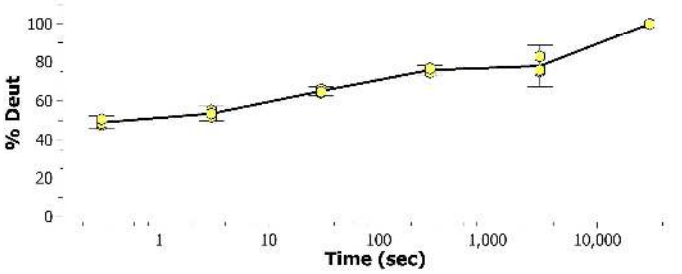

P04196 493-522: NQPFPQSVSESCPGKFKSGFPQVSMFFTHT (#121)

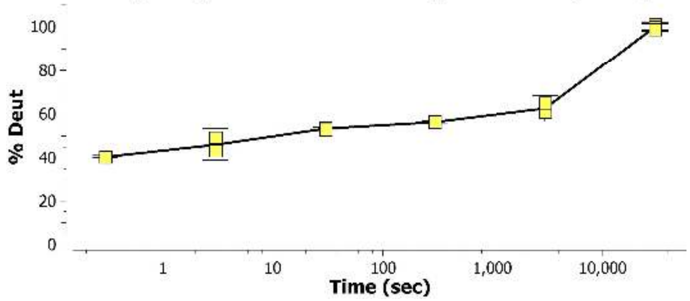

P04196 498-522: QSVSESCPGKFKSGFPQVSMFFTHT (#122)

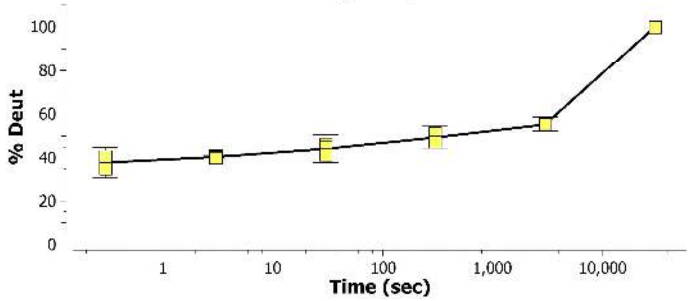

P04196 502-514: ESCPGKFKSGFPQ (#123)

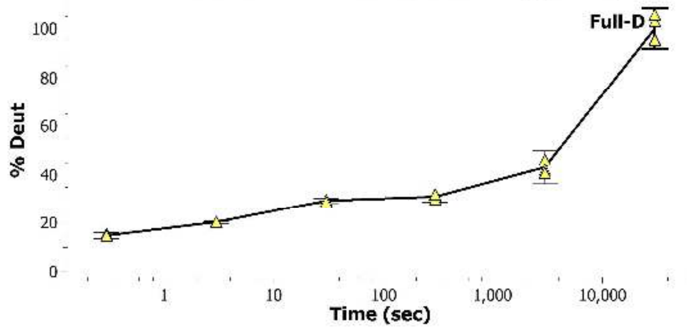

P04196 509-517: KSGFPQVSM (#124)

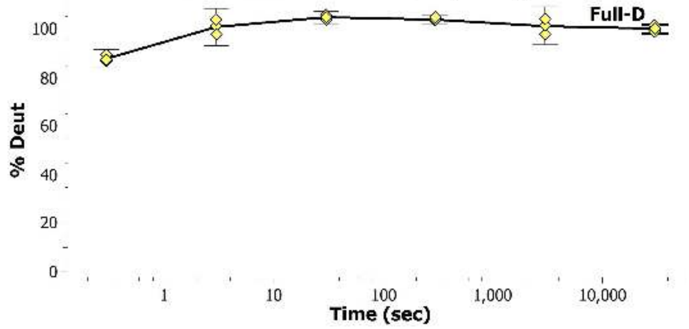

P04196 509-518: KSGFPQVSMF (#125)

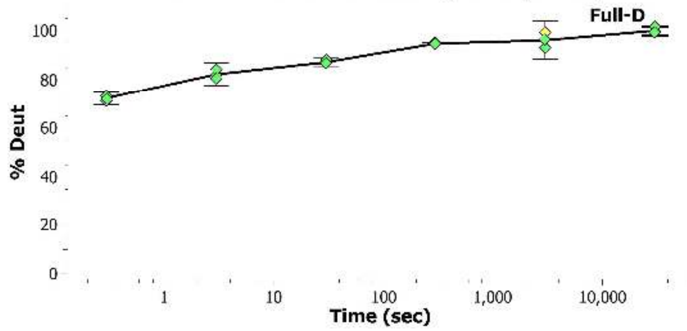

P04196 510-517: SGFPQVSM (#126)

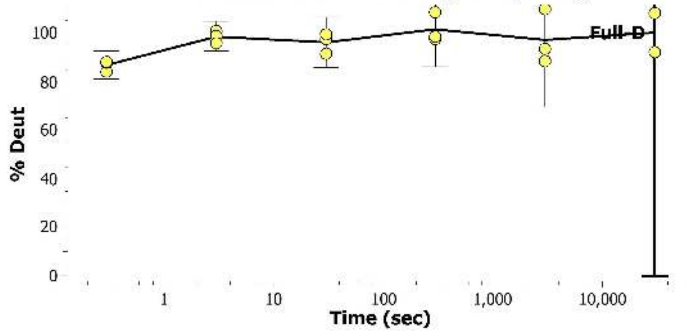

P04196 511-517: GFPQVSM (#127)

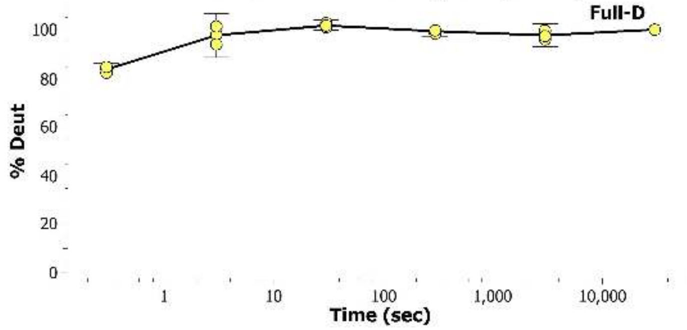

P04196 513-518: PQVSMF (#128)

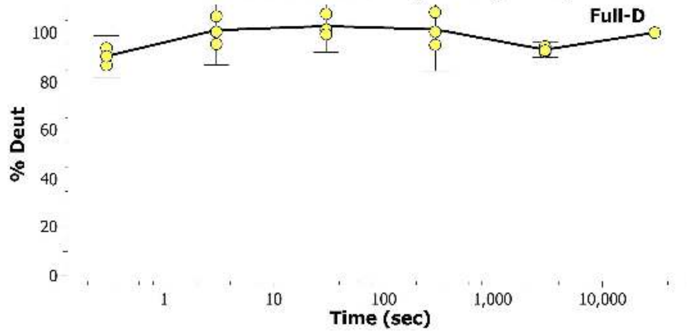

P04196 518-525: FFTHTFPK (#129)

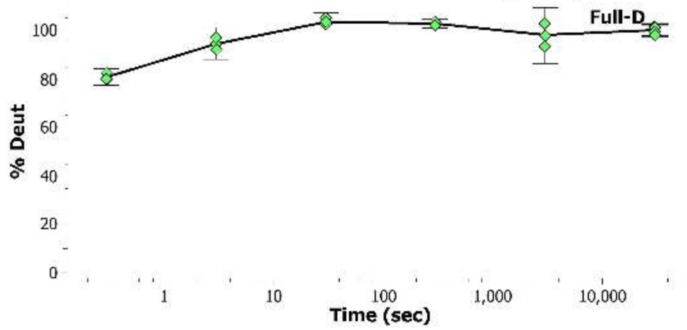

P04196 519-525: FTHTFPK (#130)

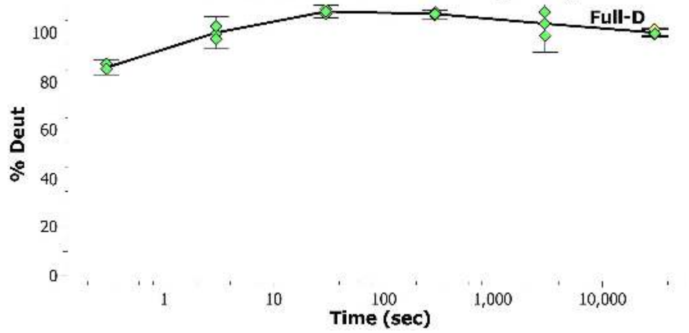

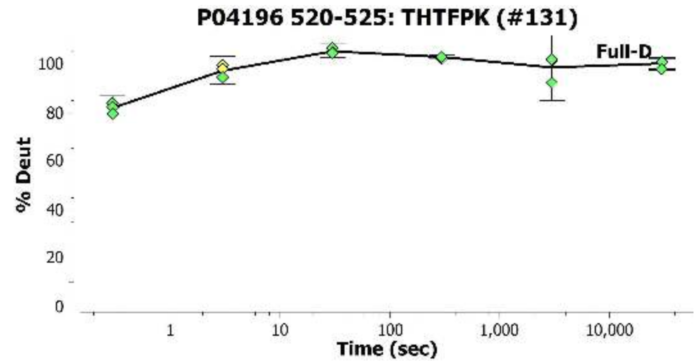

Figure S3. D_2O uptake plots for all HRG peptides. Error bars represent one standard deviation from 3 independent D_2O exchange reactions.
